# Optimal exercise dose for glycemic control in prediabetes across different exercise types

**DOI:** 10.1016/j.isci.2025.113980

**Published:** 2025-11-10

**Authors:** Ruixiang Yan, Yueming Li, Shiqi Jia, Jiaxin He, Gesheng Lin, Weifeng Huang, Jian Sun, Duanying Li

**Affiliations:** 1 School of Athletic Training, Guangzhou Sport University, Guangzhou, Guangdong, China; 2 Guangdong Provincial Key Laboratory of Human Sports Performance Science, Guangzhou Sport University, Guangzhou, Guangdong, China; 3 Key Laboratory of Human-Computer Intelligent Interaction for Athletic Performance and Health Promotion, Guangzhou Sport University, Guangzhou, Guangdong, China

**Keywords:** Kinesiology, Human metabolism

## Abstract

This study assessed the optimal exercise modality and dose for HbA1c reduction in individuals with prediabetes, taking baseline BMI into account. We searched six databases and conducted pairwise, network, and dose-response meta-analyses. Thirty-nine RCTs (3421 participants) showed that all exercise modalities significantly reduced HbA1c compared with controls, with HIIT showing the greatest effect, followed by combined, aerobic, resistance, and mind-body training. Dose-response analysis identified an optimal dose of 850 METs·min/week, whereas the minimum effective dose varied according to BMI and exercise type. These findings indicate that exercise improves HbA1c in prediabetes through a nonlinear dose-response and support BMI-stratified, individualized exercise prescriptions to optimize glycemic control.

## Highlights

• Exercise may improve HbA1c levels in individuals with prediabetes
• A J-shaped nonlinear relationship exists between exercise dose and HbA1c improvement
• The optimal exercise dose for improving HbA1c in prediabetes is about 850 MET· min/week
• The minimum effective dose varies by baseline BMI and exercise modality

## Graphical abstract

**Figure:**
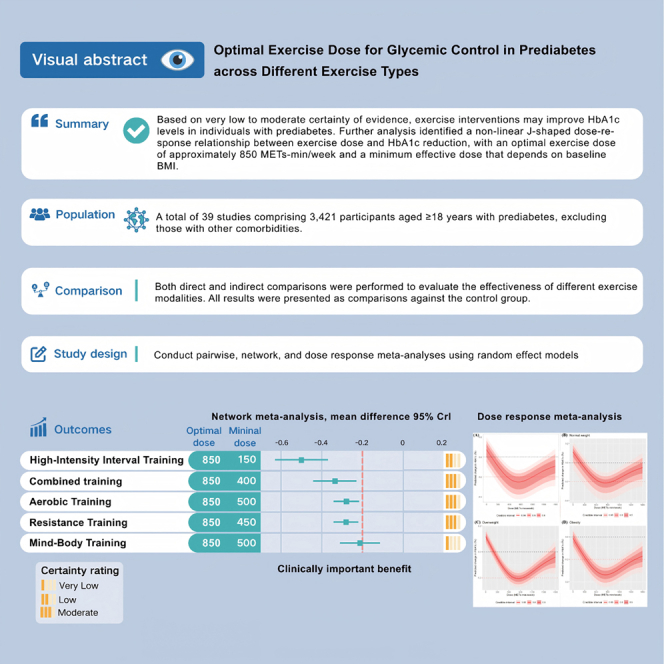

## Introduction

Prediabetes is when blood glucose or glycated hemoglobin (HbA1c) levels are elevated but do not meet the diagnostic criteria for type 2 diabetes (T2D). It represents a critical intervention window during which diabetes progression can still be prevented or reversed.^1^^,^^2^ According to the International Diabetes Federation (IDF) 2019 report, approximately 374 million adults worldwide have prediabetes, accounting for 7.5% of the adult population. In 2045, this number will increase to 548 million (8.6%).^3^ Without timely intervention, 5%–10% of individuals with prediabetes progress to T2D annually, and approximately 70% eventually develop T2D.^4^ This poses a significant public health and healthcare burden.

HbA1c is widely used to monitor glycemic control in T2D patients, reflecting average blood glucose levels over the past 2–3 months.^5^ It is closely associated with the risk of cardiovascular disease and microvascular complications.^6^ Studies have shown that a 1% reduction in HbA1c decreases the risk of cardiovascular events by 15%–20% and the risk of microvascular complications by 37%.^7^ Thus, controlling and reducing HbA1c levels is crucial for preventing T2D and its complications. As a core component of lifestyle interventions, exercise improves glycemic control and insulin sensitivity, enhances body composition, reduces blood pressure and lipid levels, and mitigates other cardiovascular risk factors.^8^^,^^9^ Current guidelines recommend at least 150 min per week of moderate-intensity physical activity as the first-line non-pharmacological strategy for managing and preventing T2D and cardiovascular diseases.^10^

Existing evidence suggests a significant nonlinear dose-response relationship between exercise and HbA1c reduction; additional health benefits may also be observed when exercise dose exceeds current guideline recommendations.^11^^,^^12^ Nevertheless, most existing studies focus on individuals with diagnosed T2D, while the optimal exercise dose and modality for prediabetes remain unclear.^11^^,^^12^ Since the primary intervention goal for prediabetes is to delay disease progression rather than reverse symptoms, the optimal exercise strategy may differ from that for individuals with established T2D. Recently, Zhang et al.^13^ conducted a dose-response meta-analysis confirming the effectiveness of aerobic training (AT), resistance training (RT), and combined training (CT) in reducing HbA1c, and proposed the respective optimal doses. However, their analysis did not assess whether these interventions achieved reductions that met the minimal clinically important difference (MCID) threshold. Moreover, previous studies did not include newer interventions such as high-intensity interval training (HIIT) and mind-body exercise (MBT), which are emphasized in the latest guidelines from the American Diabetes Association (ADA).^14^^,^^15^^,^^16^ As a result, the most effective type and dose of exercise for individuals with prediabetes remains unclear.

Furthermore, body mass index (BMI) is an important predictor of T2D risk.^17^^,^^18^ Elevated BMI may make glycemic control more challenging and substantially increase the risk of cardiovascular and other metabolic diseases.^17^^,^^18^ Although exercise interventions have been shown to improve glycemic control in individuals with prediabetes, high-quality evidence on the optimal exercise dose and the effectiveness of exercise across different BMI levels, particularly in improving HbA1c and other metabolic indicators, remains limited.^19^ This limitation indicates that the principle of “risk-stratified management” has not yet been fully integrated into exercise intervention research.^20^ Although the ADA provides general exercise recommendations for the overall population, developing evidence-based, BMI-stratified exercise prescriptions remains an urgent priority for both clinical practice and guideline development.

To address these gaps, we conducted pairwise, network, and dose-response meta-analyses within a Bayesian framework, incorporating a minimally contextualized framework based on the MCID to systematically evaluate the effectiveness of different exercise modalities on HbA1c levels in individuals with prediabetes. We further explored the dose-response relationship between exercise volume and changes in HbA1c, with stratified analyses based on baseline BMI. Adherence and adverse events were also assessed to identify the most clinically meaningful and real-world feasible exercise strategies.

## Results

### Literature selection and study characteristics

**Figure 1:**
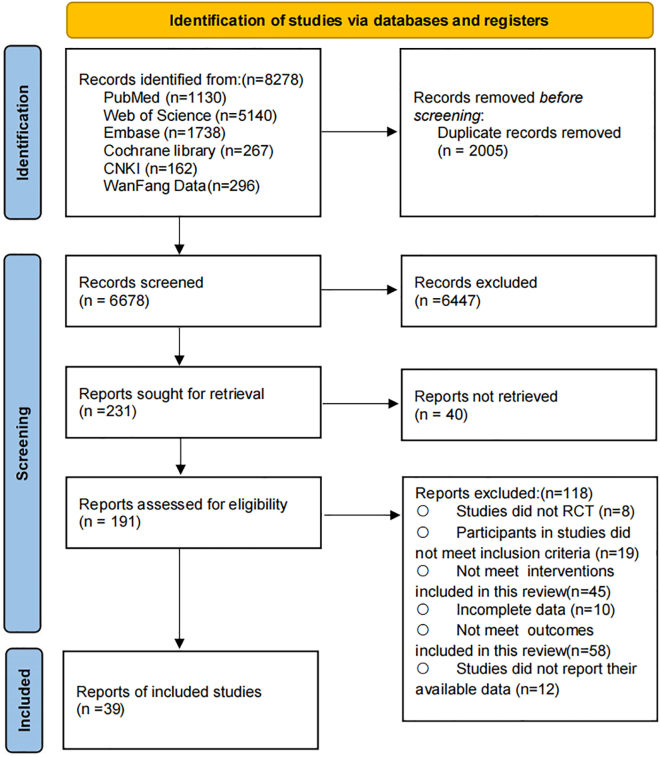
PRISMA Flow diagram of the search process for studies

A total of 8,278 potential records were identified through the systematic search. After removing duplicates, 6,678 articles remained for title and abstract screening. Among them, 191 articles met the criteria for full-text review. Ultimately, 39 studies were included in this systematic review and meta-analysis, involving 3421 participants, of whom 45.9% were male. The mean age of participants was 55.82 years, and the mean BMI was 26.85. The included studies were conducted across 12 countries, with the majority from China (*n* = 26, 66.7%), followed by India (*n* = 2, 5.1%), Korea (*n* = 2, 5.1%), and single studies each from Iran, Germany, Sweden, Spain, USA, Brazil, Egypt, Canada, and Finland (all *n* = 1, 2.6% each). The complete screening and selection process is presented in Figure 1, and the detailed characteristics of the included studies are provided in Table S12.

### Risk of bias, certainty of evidence, and consistency

**Figure 2:**
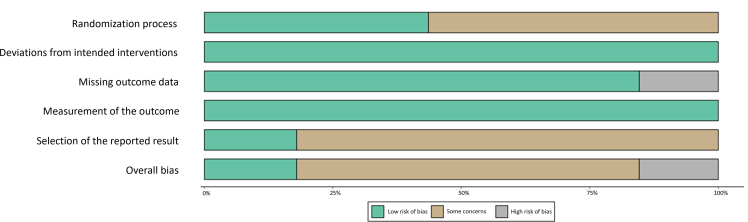
The overall risk of bias is presented as a percentage of each risk of bias item across all included studies

Overall, seven studies (17.9%) were classified as having a low risk of bias, 27 studies (69.2%) were categorized as having an unclear risk of bias, and five studies (12.8%) were deemed to have a high risk of bias (Figure 2). The detailed risk of bias assessment for each study is presented in Table S2.1.

After evaluating the certainty of evidence using the CINeMA framework, we found that most pairwise comparisons had very low to moderate certainty (Table 1). All networks met the transitivity assumption, ensuring the validity of indirect comparisons (Table S8.1). Additionally, funnel plot analysis and Egger’s test (*p* = 0.77) did not reveal substantial asymmetry, indicating no significant publication bias (Figure S9.1).

**Table 1:** CINeMA results of HbA1c

Comparison	Within study bias	Reporting bias	Indirectness	Imprecision	Heterogeneity	Incoherence	Confidence rating
AT: CT	Some concerns	No concerns	No concerns	Major concerns	No concerns	No concerns	Very low
AT: HIIT	Some concerns	No concerns	No concerns	No concerns	Major concerns	Major concerns	Very low
AT: MBT	Some concerns	No concerns	No concerns	Major concerns	No concerns	No concerns	Very low
AT: RT	Some concerns	No concerns	No concerns	Major concerns	No concerns	No concerns	Very low
AT: UC	Some concerns	No concerns	No concerns	No concerns	No concerns	No concerns	Moderate
CT: HIIT	Some concerns	No concerns	No concerns	No concerns	Major concerns	No concerns	Very low
CT: RT	Some concerns	No concerns	No concerns	Major concerns	No concerns	Major concerns	Very low
CT: UC	Some concerns	No concerns	No concerns	No concerns	No concerns	No concerns	Moderate
HIIT: UC	Some concerns	No concerns	No concerns	No concerns	No concerns	Major concerns	Very low
MBT: UC	Some concerns	No concerns	No concerns	No concerns	Major concerns	No concerns	Very low
RT: UC	Some concerns	No concerns	No concerns	No concerns	No concerns	No concerns	Moderate
Indirect evidence only							
CT: MBT	Some concerns	No concerns	No concerns	Major concerns	No concerns	Major concerns	Very low
HIIT: MBT	Some concerns	No concerns	No concerns	No concerns	Major concerns	No concerns	Very low
HIIT: RT	Some concerns	No concerns	No concerns	No concerns	Major concerns	No concerns	Very low
MBT: RT	Some concerns	No concerns	No concerns	Major concerns	No concerns	Major concerns	Very low

HIIT, High-Intensity Interval Training; CT; Combined Training; AT; Aerobic Training, RT, Resistance Training; MBT, Mind-Body Training.

For the model fit assessment of HbA1c outcomes, we compared the global consistency model with the inconsistency model. The results showed that the DIC value for the consistency model was 236.1, while the DIC value for the inconsistency model was 237, indicating a similar model fit and no statistically significant global inconsistency. In the local consistency assessment, which evaluates the agreement between direct and indirect evidence, the node-splitting method revealed significant inconsistencies in the comparisons of HIIT vs. UC (*p* = 0.013), HIIT vs. AT (*p* = 0.015), and RT vs. CT (*p* < 0.01). As a result, these comparisons were downgraded during the certainty of evidence assessment. The heterogeneity analysis indicated no significant heterogeneity across the network (τ^2^ = 0.016) (Table S3.1).

### Pairwise meta-analyses

This study included 11 pairwise comparisons, demonstrating that, compared with the control group, HIIT significantly reduced HbA1c levels (MD = −0.78%; 95% CI: −1.16 to −0.4). The effect of CT (MD = −0.36%; 95% CI: −0.45 to −0.27), AT (MD = −0.28%; 95% CI: −0.35 to −0.27), RT (MD = −0.30%; 95% CI: −0.39 to −0.21), and MBT (MD = −0.19%; 95% CI: −0.27 to −0.11) also showed significant reductions in HbA1c (Appendix 6; Table 2).

**Table 2:** League table of direct and network comparisons of the effects of different exercise interventions on HbA1c

AT	0.07 (−0.03, 0.17)	0.13 (−0.05, 0.2)	−0.04 (−0.12, 0.05)	−0.04 (−0.11, 0.03)	−0.28 (−0.35, −0.22)
0.06 (−0.05, 0.17)	**CT**	0.1 (−0.21, 0.41)	–	0.13 (0, 0.26)	**−0.36 (-0.45, -0.27)**
**0.22 (0.09, 0.34)**	0.16 (0, 0.31)	**HIIT**	–	–	**−0.78 (-1.16,-0.4)**
−0.07 (−0.16, 0.02)	−0.13 (−0.27, 0.01)	**−0.29 (-0.44, -0.14)**	**MBT**	–	**−0.19 (-0.27,-0.11)**
0 (−0.07, 0.07)	−0.06 (−0.18, 0.06)	**−0.22 (-0.36, -0.08)**	0.07 (−0.04, 0.18)	**RT**	**−0.30 (-0.39, -0.21)**
**−0.28 (-0.33, -0.22)**^a^	**−0.34 (-0.45, -0.23)**^a^	**−0.5 (-0.62, -0.36)**^a^	**−0.21 (-0.29, -0.12)**	**−0.28 (-0.35, -0.21)**^a^	**UC**

Table 2 presents the direct and network effects of different exercise modalities on HbA1c, with all effect sizes expressed as mean difference (MD) and 95% credible intervals (CrI). The network meta-analysis results are displayed in the lower-left half, while the results of the pairwise meta-analysis are shown in the upper-right half. Bolded cells indicate statistically significant results. According to the CINeMA framework for network meta-analysis, the certainty of evidence for our comparisons is included in the league table.

HIIT, High-Intensity Interval Training; CT; Combined Training; AT; Aerobic Training, RT, Resistance Training; MBT, Mind-Body Training.

a Denotes moderate certainty.

### Network meta-analyses

**Figure 3:**
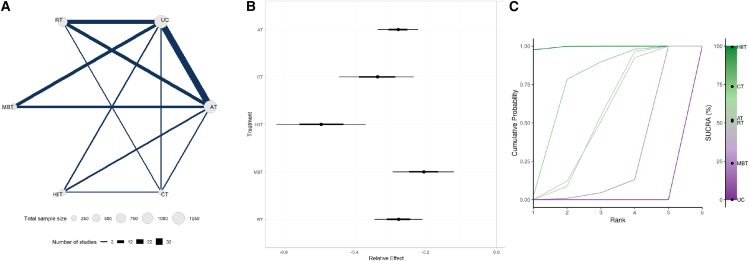
Network meta-analysis of different exercise interventions on HbA1c (A) Network diagram showing direct and indirect comparisons among interventions; (B) Forest plot of relative treatment effects on HbA1c levels. Data are presented as MD with 95% CrIs; (C) Bayesian ranking plot showing cumulative ranking probabilities and SUCRA values.

The network meta-analysis results showed that, compared with the control group, all exercise modalities effectively reduced HbA1c levels: HIIT (MD = −0.5%, 95% CrI: −0.62 to −0.36, SUCRA 99%, very low confidence of evidence), CT (MD = −0.34%, 95% CrI: −0.45 to −0.23, SUCRA 74%, moderate confidence of evidence), AT (MD = −0.28%, 95% CrI: −0.33 to −0.22, SUCRA 52%, moderate confidence of evidence), RT (MD = −0.28%, 95% CrI: −0.36 to −0.21, SUCRA 51%, moderate confidence of evidence), and MBT (MD = −0.21%, 95% CrI: −0.29 to −0.12, SUCRA 24%, very low confidence of evidence) (Table 2). Further comparisons indicated that HIIT was significantly more effective in reducing HbA1c than AT (MD = −0.22%, 95% CrI: −0.34 to −0.09, very low confidence of evidence), RT (MD = −0.22%, 95% CrI: −0.34 to −0.09, very low confidence of evidence), and MBT (MD = −0.29%, 95% CrI: −0.44 to −0.14, very low confidence of evidence) (Appendix 4). Figure 3 presents the key evidence for specific exercise modalities. The results of the minimally contextualized framework are in Table 3.

**Table 3:** The results of the minimally contextualized framework

Outcomes, certainty of the evidence, and classification of intervention	Intervention	Intervention vs. Usual care	Bayesian SUCRA	Minimum clinically effective dose	Adherence
High certainty (moderate to high certainty evidence)					
Category 2: among the most effective	CT	−0.34 (−0.45 to −0.23)	74%	850	2.5 (0.49–13)
	AT	−0.28 (−0.33 to −0.22)	52%	–	1.6 (0.74–3.3)
	RT	−0.28 (−0.36 to −0.21)	51%	–	2.2 (0.93–5.4)
Low certainty (low to very low certainty evidence)					
Category 2: among the most effective	HIIT	0.5 (−0.62 to −0.36)	99%	350	0.9 (0.15–5.1)
Category 1: intermediately effective	MBT	−0.21 (−0.29 to −0.12)	24%	–	1.1 (0.25–4.5)

HIIT, High-Intensity Interval Training; CT; Combined Training; AT; Aerobic Training, RT, Resistance Training; MBT, Mind-Body Training.

The results of the network meta-analysis showed no statistically significant differences in adherence between exercise interventions and the control group (Figure S4.3). Among all interventions, HIIT had the lowest adherence (OR = 0.91, 95% CI: 0.15 to 5.1), while CT had the highest (OR = 2.5, 95% CI: 0.49 to 13). The remaining interventions ranked as follows: RT (OR = 2.2, 95% CI: 0.93 to 5.4), AT (OR = 1.6, 95% CI: 0.74 to 3.3), and MBT (OR = 1.1, 95% CI: 0.25 to 4.5).

### Dose-response relationships between exercise and HbA1c

**Figure 4:**
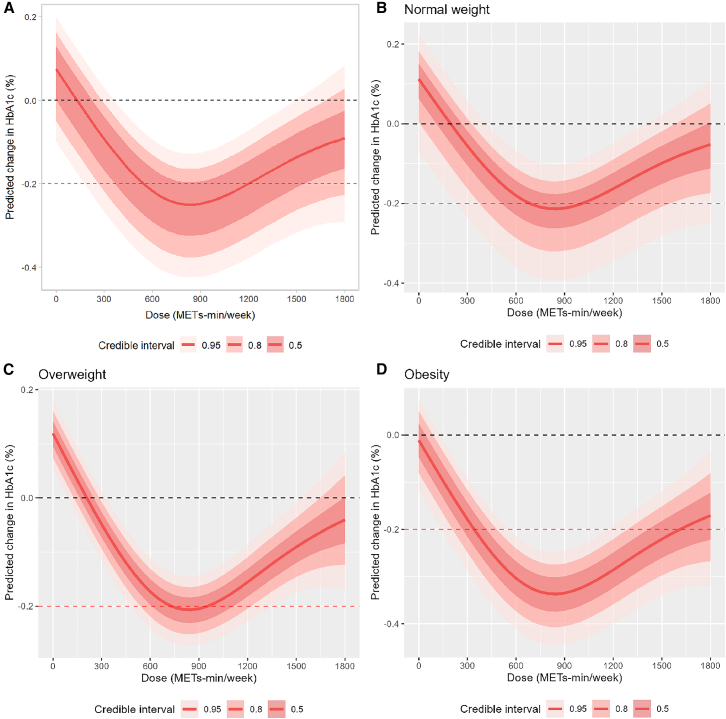
Dose-response relationship between weekly exercise dose and HbA1c levels in prediabetes (A) overall population; (B) normal weight (BMI <24); (C) overweight (24 ≤ BMI <28); (D) obesity (BMI ≥28).

A nonlinear J-shaped dose-response relationship was observed between exercise dose and reductions in HbA1c, and the optimal exercise dose was estimated to be 850 METs·min/week (Figure 4A). At this dose, the predicted reductions in HbA1c were −0.20% to −0.26% in individuals with normal weight, −0.16% to −0.33% in those who were overweight, and −0.34% to −0.35% in those with obesity (Table S7.1). Predicted changes in HbA1c were modeled and visualized after adjusting for baseline BMI and stratifying according to the Chinese BMI classification (Figure S7.1). Following BMI adjustment, statistically significant reductions in HbA1c were observed across all BMI categories at the optimal dose; however, only individuals with obesity demonstrated reductions that surpassed the MCID (MD = −0.339%; 95% CrI, −0.440 to −0.238) (Table 4). Among individuals with obesity, the minimum effective dose required to achieve a clinically meaningful reduction in HbA1c was estimated at 550 METs·min/week, suggesting that clinically relevant benefits may be achieved at lower exercise doses in this subgroup (Figure 4D). By contrast, even at the optimal dose, individuals with normal weight or overweight did not achieve clinically meaningful reductions (Figures 4B and 4C). The estimated effects per 1-unit increase in BMI and their associated uncertainty intervals are summarized in Table S8.1. In addition to exercise dose, we also observed a nonlinear association between exercise intensity and reductions in HbA1c (Figure S7.5). At a fixed weekly duration of 150 min, HbA1c reduction became statistically significant at an intensity of approximately 2.8 METs, whereas a reduction exceeding the MICD was observed at around 8.3 METs. When classified by conventional categories, low-intensity activity (1.6–2.9 METs) showed no significant effect (MD = −0.017%; 95% CrI: −0.118 to 0.083), while moderate-intensity (3.0–5.9 METs; MD = −0.15%; 95% CrI: −0.236 to −0.063) and vigorous-intensity activity (≥6.0 METs; MD = −0.28%; 95% CrI: −0.400 to −0.160) were associated with progressively greater reductions. These results suggest that achieving improvements in HbA1c may require surpassing a minimum effective intensity threshold.

**Table 4:** Minimal effective and optimal exercise doses according to BMI category

BMI Category	Minimal Dose (METs min/week)	MCFB % HbA1c (95% CrI)	Optimal Dose (METs min/week)	MCFB % HbA1c (95% CrI)	Physical activity intensity (METs)	Minimum recommended accumulation (min/weeks)	Optimal recommended accumulation (min/weeks)
Normal weight	500^a^	−0.157 (−0.300,-0.015)	850^a^	−0.224 (−0.366,-0.082)	Light: 1.6–2.9	172–313	293–531
					Moderate:3.0–5.9	85–166	144–283
					Vigorous:≥6.0	83	142
Overweight	400^a^	−0.139 (−0.260,-0.019)	850^a^	−0.208 (−0.273,-0.142)	Light: 1.6–2.9	138–250	293–531
					Moderate: 3.0–5.9	68–133	144–283
					Vigorous: ≥6.0	67	142
Obesity	550^b^	−0.31 (−0.407,-0.213)	850^b^	−0.339 (−0.440,-0.238)	Light: 1.6–2.9	190–344	293–531
					Moderate: 3.0–5.9	93–183	144–283
					Vigorous: ≥6.0	92	142

Recommended accumulation values are calculated by converting total energy expenditure (MET·min/week) into equivalent weekly minutes using Compendium-based intensity categories (light 1.6–2.9, moderate 3.0–5.9, vigorous ≥6.0 METs). These values are provided for reference. Our analysis indicates that HbA1c reduction became statistically significant only at intensities >2.8 METs, suggesting that very low-intensity activities within the “light” range may not produce meaningful glycemic benefits.

MCFB % HbA1c. Mean Change from Baseline % HbA1c.

a Indicates that MCFB is statistically significant but not clinically meaningful because the 95% Cr includes values greater than −0.20%.

b Indicates that MCFB % HbA1c (95% CrI) is clinically and statistically significant.

In analyses comparing exercise modalities, the optimal dose remained consistent at 850 METs·min/week irrespective of baseline BMI adjustment (Figure S7.4). In unadjusted analyses, HIIT (MD = −0.52%; 95% CrI, −0.598 to −0.443) and CT (MD = −0.262%; 95% CrI, −0.323 to −0.201) were the most efficacious interventions for reducing HbA1c (Table 5; Figure S7.1). HIIT produced clinically meaningful reductions at doses as low as 350 METs·min/week, whereas CT attained clinical significance only at the optimal dose of 850 METs·min/week. After adjusting for BMI, the minimum effective dose of HIIT demonstrated clinically meaningful effects across all BMI categories, whereas CT yielded significant clinical benefits only among individuals with obesity at the optimal dose (Table S7.2; Figure S7.3). The estimated effects per 1-unit increase in BMI and their corresponding uncertainty intervals are presented in Table S13.

**Table 5:** Minimal effective and optimal doses for each exercise type

Type of exercises	Minimal Dose (METs min/week)	MCFB % HbA1c (95% CrI)	Optimal Dose (METs min/week)	MCFB % HbA1c (95% CrI)	Code	Mets	Minimum recommended accumulation (min/weeks)	Optimal recommended accumulation (min/weeks)
HIIT	350^b^	−0.306 (−0.371,-0.24)	850^b^	−0.52 (−0.598,-0.443)	02040	7	50	121
					02015	11	32	77
CT	850^b^	−0.262 (−0.323,-0.201)	850^b^	−0.262 (−0.323,-0.201)	0205417346	3.2	266	266
					0205217358	4.9	173	173
					0205017364	6.4	133	133
AT	500^a^	−0.087 (−0.14,-0.034)	850^a^	−0.2 (−0.239,-0.16)	17346	2.8	179	304
					17358	4.8	104	177
					17364	6.8	74	125
RT	450^a^	−0.067 (−0.123,-0.01)	850^a^	−0.211 (−0.255,-0.167)	02054	3.5	129	243
					02052	5	90	170
					02050	6	75	142
MBT	500^a^	−0.07 (−0.131,-0.008)	850^a^	−0.183 (−0.251,-0.114)	15670	3.3	152	258
					02160	4	125	213
					15674	6	83	142

Recommended accumulation values were derived from the Compendium of Physical Activities by converting total energy expenditure (MET·min/week) into equivalent weekly minutes at each intensity category. These values are provided for reference. Our analysis indicates that HbA1c reduction became statistically significant only at intensities >2.8 METs, suggesting that very low-intensity activities within the “light” category may not provide meaningful glycemic benefits.

HIIT, High-Intensity Interval Training; CT; Combined Training; AT; Aerobic Training, RT, Resistance Training; MBT, Mind-Body Training.

MCFB % HbA1c. Mean Change from Baseline % HbA1c.

a Indicates that MCFB is statistically significant but not clinically meaningful because the 95% Cr includes values greater than −0.20%.

b Indicates that MCFB % HbA1c (95% CrI) is clinically and statistically significant.

### Meta-regressions and sensitivity analyses

To further assess the robustness of the results, we conducted meta-regression analyses to explore potential sources of heterogeneity in all outcome measures. The factors included in the regression analysis were publication year, mean age, proportion of male participants, baseline BMI, baseline HbA1c, sample size, exercise period, frequency, and session duration. The results indicated that HbA1c reduction was significantly associated with exercise frequency (β = −0.10; 95% CI: −0.002 to −0.2046), while no significant associations were found with other covariates (Table S10.1). Additionally, after adjusting all potential sources of heterogeneity to their median values and reanalyzing the data, the findings remained consistent with the original analysis, further confirming the robustness of the sensitivity analyses. We also conducted two additional sensitivity analyses: one under different assumed pre-post correlations (r = 0.3, 0.5, and 0.7) and another excluding studies at high risk of bias. Both analyses yielded results consistent with the primary findings, supporting the stability of our conclusions (Appendix 11).

### Adverse events

Among the included studies, a total of three reported exercise-related adverse events. In the AT group (135 participants), four adverse events were reported across three studies, including one sprain, one cardiovascular event, one fall, and one case of hypoglycaemia. In the HIIT group (94 participants), two studies reported seven adverse events in total, including six soft tissue injuries, one cardiovascular event, and one case of knee pain. In the RT group (42 participants), one study reported three adverse events, including two muscle strains and one case of hypoglycaemia. Overall, the reported adverse events were primarily mild to moderate in severity, with a low incidence rate, indicating good overall tolerability of the exercise interventions.

## Discussion

### Principal findings

This study systematically evaluated the effects of different exercise modalities on glycemic control in individuals with prediabetes and examined the dose-response relationship between exercise volume and changes in HbA1c. We identified a nonlinear J-shaped association, in which an exercise volume of approximately 850 METs·min/week yielded the greatest improvement in HbA1c. In contrast, higher volumes did not cf. additional clinical benefits. Importantly, baseline BMI was inversely associated with the minimum effective dose required to achieve a clinically meaningful reduction in HbA1c. Among individuals with obesity, as little as 550 METs·min/week was sufficient to surpass the MCID threshold. By contrast, individuals with normal weight or mild overweight exhibited statistically significant reductions, but these did not meet the predefined MCID. Furthermore, all exercise modalities were associated with significant HbA1c reductions, although the magnitude of effect varied. Among them, CT led to clinically meaningful improvements, supported by moderate-certainty evidence, especially among individuals with obesity. HIIT also demonstrated potential for clinically meaningful effects across BMI subgroups; however, the very low certainty of evidence substantially limits confidence in its actual effect and calls for further validation. Collectively, these findings establish clinically actionable, BMI-stratified thresholds for exercise prescriptions in prediabetic populations. They support a shift from generic recommendations toward individualized, evidence-based exercise strategies, informing both clinical decision-making, and future updates to diabetes prevention guidelines.

### Comparison with other studies

According to current ADA recommendations, individuals are advised to perform at least 150 min of moderate-intensity physical activity per week to prevent diabetes. In contrast, our analysis identified an optimal exercise dose of 850 METs·min/week, equivalent on average to approximately 188 min of moderate-intensity aerobic activity (ranging from 144 to 283 min per week, depending on exercise intensity between 3 and 6 METs) or about 121 min of high-intensity aerobic activity per week at an estimated intensity of 7 METs. These findings suggest that achieving clinically meaningful improvements in HbA1c may require a greater overall exercise volume than currently recommended by existing guidelines. Similarly, previous studies have explored the relationship between exercise dose and HbA1c in T2D patients. For example, Gallardo-Gómez et al.^12^ found that 1,100 METs·min/week was the optimal dose for HbA1c improvement, while Liang et al.^11^ reported that the marginal effect started to decline at 1,300 METs·min/week. These optimal doses are significantly higher than ADA recommendations. Notably, while the optimal exercise dose for prediabetes and T2D patients exceeds ADA recommendations, the required dose for prediabetes patients is lower than for T2D patients. This difference may be attributed to prediabetes patients still retaining partially functional β cells, making exercise interventions more effective in improving metabolic function.^4^ In contrast, T2D patients, due to progressive β cell deterioration and impaired peripheral insulin signaling, may require more sustained and long-term exercise training to restore metabolic homeostasis.^21^ Recently, Zhang et al.^13^ supported this view by identifying 870 METs·min/week as the optimal dose of AT for improving HbA1c, which is consistent with our findings. However, their study primarily focused on whether the statistical effect was significant and did not evaluate whether the improvement exceeded the threshold of the MCID. Moreover, they failed to identify precise optimal doses for RT and CT. In contrast, our study incorporated a broader range of exercise modalities, including HIIT, CT, and MBT, performed stratified analyses based on baseline BMI, and applied MCID as a threshold to determine clinical relevance. These additions enhanced the interpretability and applicability of our findings in clinical practice.

This study suggests that HIIT may have greater potential for improving glycemic control and may achieve clinically meaningful reductions in HbA1c across different BMI categories. Moreover, our findings indicate that HIIT could be more effective than other exercise modalities in lowering HbA1c, suggesting that the type of exercise itself may play an important role in glycemic improvement. These observations contrast with the conclusions of Gallardo-Gómez et al.^12^ and Liang et al.,^11^ who proposed that exercise dose may be more critical than exercise type. However, as this conclusion is drawn from very low-certainty evidence, the actual effect remains highly uncertain. Caution is therefore warranted in clinical interpretation, and further high-quality randomized controlled trials are required to validate these findings. Furthermore, the minimum effective dose for HIIT was 350 METs·min/week, requiring only 32 min of high-intensity exercise per week to achieve a clinically significant HbA1c reduction, making it particularly suitable for time-constrained individuals. Previous research has also demonstrated that HIIT outperforms continuous aerobic exercise in improving insulin sensitivity and glycemic control in healthy individuals. HIIT has been shown to activate skeletal muscle AMPK signaling pathways, leading to a 30% increase in insulin sensitivity within 24 h.^22^ However, whether exercise intensity provides additional glycemic benefits in T2D patients remains controversial. Studies have shown that HIIT induces either similar^23^^,^^24^ or greater^25^^,^^26^^,^^27^ HbA1c reductions when energy expenditure is matched compared to low to moderate-intensity continuous aerobic exercise. While high-intensity training effectively enhances insulin sensitivity and endothelial function, additional clinical trials are needed to determine optimal intensity levels for different health outcomes in prediabetes patients.^24^

Although HIIT offers time efficiency and metabolic benefits, its potential risk of musculoskeletal injuries should be considered.^28^ Notably, prediabetes patients often present with sedentary behavior and obesity, which may limit their physical function. In large-scale clinical trials, such as the Diabetes Prevention Program (DPP)^29^ and Finnish Diabetes Prevention Study (FDPS),^30^ individuals with multiple comorbidities and physical limitations were excluded, as these conditions could interfere with exercise adherence. This selection bias may overestimate the real-world applicability of HIIT.^31^ Notably, this study observed that adherence to HIIT was significantly lower than that of other exercise modalities, as reflected by a higher dropout rate. Several participants reported adverse events, including muscle strain and cardiovascular reactions, suggesting that despite HIIT’s substantial metabolic benefits, its safety and long-term sustainability warrant particular attention. This may be due to the inherently high intensity and cardiovascular demands of HIIT, which could limit its feasibility, especially among individuals with prediabetes who are obese or have limited physical capacity. Therefore, although HIIT demonstrated the most pronounced statistical effects, the very low certainty of the evidence and uncertainties regarding its long-term feasibility warrant cautious evaluation of its target population and implementation strategies in clinical practice. In real-world clinical settings, interventions with higher adherence, better safety profiles, and greater long-term feasibility are often more conducive to achieving sustainable glycemic management, and exercise prescriptions should be individualized according to each patient’s health status and physical fitness, with careful consideration of the balance between potential efficacy and associated risks to ensure both safety and feasibility.

In addition to HIIT, CT probably effectively reduces HbA1c levels in individuals with prediabetes, with a reduction regarded as clinically meaningful according to the predefined MCID, supported by moderate-certainty evidence. Previous studies have also reported that CT is likely to be more effective than either AT or RT alone, which is consistent with our findings.^32^^,^^33^ The potential advantage of CT may stem from the fact that AT and RT improve glycemic control through distinct physiological mechanisms, and their combination could produce synergistic effects across multiple metabolic pathways, thereby further enhancing the reduction in HbA1c.^34^ Additionally, Martins et al.^35^ reported that in individuals at high risk of developing T2D, HIIT achieved similar HbA1c reductions as CT in a shorter duration, highlighting its unique clinical advantages. Given that the evidence supporting CT is of moderate certainty, CT may represent a more reliable and applicable option in clinical practice.

Beyond traditional exercise modalities, MBT combines mindfulness, controlled breathing, and coordinated movement, and may support glycemic control primarily by alleviating psychological stress.^36^^,^^37^^,^^38^ Chronic stress is a recognized risk factor for T2D, linked to elevated cortisol levels, increased insulin resistance, and impaired glucose metabolism.^39^^,^^40^ MBT may mitigate these effects by promoting relaxation, reducing cortisol levels, and indirectly enhancing insulin sensitivity and glucose uptake.^36^^,^^37^^,^^38^ For individuals with comorbid anxiety, depression, or other psychological conditions, MBT may serve as a foundational approach offering additional benefits by addressing both metabolic and psychological aspects of prediabetes.^40^ Given its low intensity and favourable safety profile, MBT may also be appropriate for individuals with limited physical capacity. Although our findings suggest a potential benefit of MBT in reducing HbA1c, the certainty of evidence is very low and should be interpreted cautiously. Furthermore, emerging data suggest heterogeneous effects across MBT modalities. A network meta-analysis by Jia et al.^41^ reported that Tai Chi achieved the greatest HbA1c reduction, whereas Baduanjin and Yijinjing were also beneficial but less effective. Given the scarcity of high-quality trials for each practice, our analysis lacked statistical power to detect such differences. High-quality, modality-specific trials are needed to clarify comparative efficacy and inform tailored recommendations for diabetes prevention and management.

While exercise modality plays an important role, accumulating evidence indicates that exercise intensity may independently contribute to glycemic regulation. In support of this, high-intensity aerobic^42^ or resistance training^43^ has been associated with greater reductions in HbA1c and improvements in insulin sensitivity compared with moderate-intensity training. By contrast, low-intensity activity can improve postprandial and 24 h glucose levels in the short term, but consistent evidence for sustained reductions in HbA1c is lacking.^44^^,^^45^^,^^46^ Even in interventions aimed at interrupting sedentary behavior, moderate-intensity activity generally outperforms low-intensity approaches, particularly among individuals with higher BMI.^47^ Our findings add quantitative evidence to this body of work. When weekly duration was fixed at 150 min, reductions in HbA1c reached statistical significance only at intensities above about 2.8 METs, with clinically meaningful changes observed from around 8.3 METs. These findings demonstrate that activities at the lower end of the light-intensity spectrum are unlikely to deliver sustained glycemic benefits, even with sufficient duration, underscoring the necessity of achieving adequate intensity in addition to overall exercise volume. Nevertheless, low-intensity activity can still be recommended as a pragmatic strategy to interrupt sedentary time, particularly for individuals with limited exercise capacity.^48^ For sustained reductions in HbA1c, however, structured programmes of at least moderate intensity are warranted, with the progressive incorporation of higher-intensity training when feasible. Further research should assess the generalizability of these thresholds across diverse populations and exercise modalities to strengthen the evidence base for individualized exercise prescriptions.

Interestingly, our meta-regression analysis identified a significant association between exercise frequency and reductions in HbA1c, consistent with the findings of Umpierre et al.^49^ and Pai et al.,^50^ who underscored the pivotal role of frequency in glycemic control. Whereas current ADA guidelines primarily emphasize the duration and intensity of physical activity, our results suggest that increasing exercise frequency may yield greater benefits than extending session length or intensifying effort. This frequency-focused strategy also confers practical advantages. High-frequency, low-intensity exercise sessions can be more easily incorporated into daily routines, particularly for individuals constrained by work, family responsibilities, or physical limitations. Moreover, increasing exercise frequency helps interrupt prolonged sedentary behavior, which is a recognized risk factor for T2D.^51^ Collectively, these findings support the integration of exercise frequency as a central component in the design of personalized interventions, both for its metabolic benefits and its potential to enhance adherence, minimize dropout, and promote sustained improvements in glycemic outcomes.

### Clinical implications

This study revealed a nonlinear J-shaped dose-response relationship between exercise volume and reductions in HbA1c, providing new evidence to inform future updates of diabetes management guidelines. Current guidelines predominantly focus on exercise intensity and duration but lack specific, quantitative recommendations regarding optimal exercise dose.^52^ Our findings identified an optimal exercise volume of 850 METs·min/week, offering a more actionable and measurable benchmark for achieving meaningful improvements in HbA1c among individuals with prediabetes. Importantly, we observed an inverse association between BMI and the minimum effective exercise dose required to achieve a clinically meaningful reduction in HbA1c. Among individuals with obesity (BMI ≥28 kg/m^2^), a relatively modest dose of 550 METs·min/week was sufficient to achieve the MCID, suggesting that those with higher BMI derive greater metabolic benefits from a lower exercise dose. In contrast, in individuals with normal weight (BMI ≤23.9 kg/m^2^) or overweight (BMI 24.0–27.9 kg/m^2^), even the optimal dose of 850 METs·min/week resulted in statistically significant reductions in HbA1c. However, the effect size did not reach the prespecified MCID threshold. One plausible explanation is that improvements in HbA1c are partly mediated by weight loss, and the average weight reduction achievable through exercise or dietary interventions is typically smaller in normal-weight individuals compared with those who are overweight or obese.^53^^,^^54^ Nevertheless, regular exercise in normal-weight individuals with prediabetes confers broad metabolic and cardiovascular benefits, including improved insulin sensitivity, reductions in visceral adiposity, preservation or increases in lean mass, and enhanced cardiorespiratory fitness.^55^^,^^56^ These effects are particularly relevant because metabolic abnormalities in normal-weight individuals with prediabetes may arise independently of obesity.^57^^,^^58^ Accordingly, exercise should remain a cornerstone intervention for the prevention and management of prediabetes, even when HbA1c improvements do not meet the MCID threshold. From a clinical perspective, these findings underscore the need for BMI-stratified exercise prescriptions. For individuals with obesity, initiating exercise programmes at a lower volume, such as 550 METs·min/week, may improve adherence and safety, while still achieving clinically meaningful glycemic benefits. For those with normal weight or mild overweight, exercise prescriptions should extend beyond glycemic control to prioritize improvements in cardiorespiratory fitness, body composition, and cardiovascular risk profiles, thereby maximizing comprehensive metabolic gains. Furthermore, while different exercise modalities have demonstrated statistically or clinically significant effects on HbA1c.^14^^,^^15^, their effect sizes remain heterogeneous. Although the ADA currently recommends a uniform target of 150 min of moderate-intensity exercise per week (≈600 METs·min/week), our findings suggest that exercise dose-response patterns and the relative effectiveness of exercise modalities differ by BMI category. Future guidelines could build upon existing recommendations by incorporating BMI-stratified evidence and considering the differential impacts of exercise types, thereby supporting the development of more personalized and targeted exercise prescriptions in both clinical practice and public health strategies. To further enhance the clinical applicability of our findings, we referred to the 2024 Adult Compendium of Physical Activities for Adults. We converted the optimal and minimum effective exercise doses for different modalities into their equivalent weekly durations across varying intensity levels, stratified by BMI. This approach provides a more transparent and more quantifiable framework for exercise prescription, enabling clinicians to design BMI-specific, individualized exercise plans that can improve both the effectiveness and feasibility of exercise-based interventions in clinical practice.

### Strengths of the study

This study employed pairwise, Bayesian network, and dose-response meta-analytic models to comprehensively evaluate the effects of exercise on HbA1c levels. Integrating these three approaches enabled us to identify the most effective exercise modalities and to estimate their minimum effective, clinically meaningful, and optimal doses. The included studies provided detailed information on key exercise parameters, including duration, intensity, and frequency, which enabled robust dose assessments. To improve the accuracy and clinical relevance of energy expenditure estimates, warm-up and cool-down phases were incorporated into the total MET calculations. To ensure the credibility of our results, we applied the CINeMA framework to systematically assess the certainty of evidence, which ranged from very low to moderate. Based on this assessment, we adopted a minimally contextualized framework that integrates effect size thresholds (e.g., MCID) with the certainty of evidence, thereby enhancing the clinical interpretability of our findings and enabling objective, clinically relevant classification of exercise interventions. Finally, we conducted meta-regression analyses to explore potential moderating factors and performed three sensitivity analyses to confirm the robustness of our conclusions.

### Limitations of the study

Despite its strengths, this study has some limitations. First, approximately 79.5% of the included studies were conducted in Asian populations. While this improves the representativeness of our findings for Asian populations, it may limit the generalizability of the conclusions to regions with different cultural, lifestyle, and healthcare contexts. Future studies should include more geographically diverse populations to enhance the external validity of the evidence and support globally applicable exercise recommendations for individuals with prediabetes. Second, although we explored the potential moderating effect of intervention duration through meta-regression, no statistically significant association was identified. However, since HbA1c reflects long-term metabolic adaptations, the potential influence of the intervention period cannot be disregarded. Future research should investigate the interaction between weekly exercise dose and intervention period for determining the effectiveness of exercise interventions, which may help refine clinical recommendations. Third, the limited sample size of participants with BMI ≥32 kg/m^2^ introduces greater uncertainty in predictions beyond this range. Therefore, our dose-response findings should be interpreted as primarily applicable to individuals with BMI ≤32 kg/m^2^. Given the distinct metabolic characteristics and pronounced insulin resistance in this population, larger, high-quality studies are warranted to inform personalized exercise strategies aimed at optimizing glycemic control and improving overall health outcomes.^59^^,^^60^ Finally, the weekly exercise dose in this study was estimated using standardized MET values from the compendium of physical activities. This approach is widely adopted in exercise science and is frequently applied in recent high-quality systematic reviews and clinical guidelines, providing a consistent and comparable framework for synthesizing heterogeneous interventions. Nevertheless, actual energy expenditure may vary among individuals due to differences in physiological capacity, adherence, and environmental context. Future research should consider integrating objective activity monitoring such as wearable devices or indirect calorimetry to enhance the accuracy of dose estimation and to refine population-level recommendations and personalized exercise prescriptions further.

### Conclusion

This systematic review and meta-analysis suggest that exercise interventions may improve glycemic control in individuals with prediabetes, although the certainty of evidence varied across modalities. CT, AT, and RT probably improve HbA1c, supported by moderate-certainty evidence, whereas HIIT and MBT may improve HbA1c. However, the certainty of evidence is very low and the actual effect remains highly uncertain. We also identified a nonlinear J-shaped dose-response relationship between exercise dose and HbA1c improvement, with an optimal exercise dose of approximately 850 METs·min/week and a minimum effective dose that varies according to both baseline BMI and exercise modality. These findings provide a robust evidence base for developing BMI-stratified, modality-specific, evidence-based exercise prescriptions and support the formulation of individualized recommendations to optimize glycemic control and inform diabetes prevention strategies.

## Resource availability

### Lead contact

Further information and requests for resources should be directed to and will be fulfilled by the lead contact, Prof Duanying Li (liduany@gzsport.edu.cn).

### Materials availability

This study did not generate new unique reagents.

### Data and code availability

• The data used in this meta-analysis were obtained from published studies available in PubMed, Web of Science, Embase, Cochrane Library, CNKI, and Wanfang Data, and no new datasets were generated. All information regarding searched databases and used software are listed in key resources table.
• The codes supporting the current study have not been deposited in a public repository but are available from the lead contact on request.
• Any additional information required to reanalyze the data reported in this paper is available from the lead contact upon request.

## Acknowledgments

The work was supported by funding for Guangdong Provincial Philosophy and Social Sciences Regularization Project 2022 (GD22CTY09): Research on the Coordinated Development Path of International Competitiveness in Sports in the Guangdong-Hong Kong-Macao Greater Bay Area.

## Author contributions

R.Y. and Y.L. participated in the conception or design, acquisition, analysis, or interpretation of the data, and drafting and revising the manuscript. S.J. and J.H. participated in the acquisition, analysis, or interpretation of the data. G.S. participated in revising the manuscript and supervision. W.H., J.S., and D.L. participated in the acquisition, analysis, or interpretation of the data. All authors have read and approved the final version of the manuscript and agree with the order of authorship.

## Declaration of interests

The authors declare no competing interests.

## STAR★Methods

### Key resources table

**Table:** 

REAGENT or RESOURCE	SOURCE	IDENTIFIER
**Deposited data**		

Studies for Meta-analysis	PubMed,	https://www.ncbi.nlm.nih.gov/pubmed
	Web of Science database	https://www.webofscience.com
	Embase	https://www.embase.com
	Cochrane Library	https://www.cochranelibrary.com
	CNKI	https://www.cnki.net
	WanFang Date	https://www.wanfangdata.com.cn

**Software and algorithms**		

R 4.3.1	R project	https://www.r-project.org
StataSE 17.0	StataCorp LLC	https://www.stata.com

### Experimental model and study participant details

Our study does not use experimental models typical in the life sciences.

### Method details

#### Search strategy and study selection

A systematic search was conducted in PubMed, Web of Science, Cochrane (CENTRAL), Embase, CNKI, and WanFang Data databases to identify randomized controlled trials (RCTs) investigating the effects of exercise interventions on individuals with prediabetes, published from database inception to December 10, 2024. Three independent reviewers (YM, SQ, and GS) screened and selected studies that met the inclusion criteria. Disagreements were resolved through consultation with a fourth reviewer (WF). Additionally, reference lists of included studies and relevant systematic reviews were manually screened to identify potentially eligible studies. The complete search strategy is provided in Appendix 1.

#### Eligibility criteria

We applied the PICOS framework (Participants, Interventions, Comparators, Outcomes, and Study design) to determine study eligibility. Studies were included if they met all of the following criteria.

##### Population

We included studies involving individuals aged ≥18 years with prediabetes, excluding those diagnosed with diabetes, severe comorbidities, children, adolescents, or pregnant women. These groups were excluded because their diagnostic thresholds and metabolic profiles, including the lower glycaemic cut-offs in pregnancy and puberty-related changes in insulin sensitivity, differ substantially from those of non-pregnant adults. Including these populations could introduce considerable clinical and methodological heterogeneity, thereby limiting the applicability of the findings to the target population. The diagnosis of prediabetes was based on the ADA criteria and met at least one of the following conditions: fasting plasma glucose (FPG) between 5.6–6.9 mmol/L, HbA1c between 39–47 mmol/mol (5.7–6.4%), or 2-hour plasma glucose levels between 7.8–11.0 mmol/L during an oral glucose tolerance test (OGTT)^61^ (Table 6)

**Table:** Diagnostic criteria for prediabetes

Parameter	Test used	Prediabetes range	
		mg/dl or % (HbA1c)	mmol/l or mmol/mol(HbA1c)
IFG	FPG test	100–125	5.6–6.9
IGT	OGTT	140–199	7.8–11.0
HbA1c	HbA1c test	5.7–6.4	39–46

FPG, fasting plasma glucose; HbA1c, hemoglobin A1c; IFG, impaired fasting glucose; IGT, impaired glucose tolerance; OGTT, oral glucose tolerance test.

.

##### Intervention

The included interventions were limited to exercise-only training programs, without additional treatment modalities such as pharmacological or dietary modifications. Eligible programmes had a minimum duration of four weeks, a period widely regarded as sufficient for physiological adaptation and the attainment of sustained effects.^11^ Exercise interventions were categorized into five types: aerobic training (AT), resistance training (RT), combined training (CT), high-intensity interval training (HIIT), and mind-body training (MBT). Detailed definitions and intensity classifications for each exercise type are provided in Methods S1.

##### Comparator

Control groups included any of the five exercise modalities, health education, usual care, or wait-list controls.

##### Outcome

HbA1c (%) was used as the primary outcome measure for glycemic control.

##### Study design

Only randomized controlled trials (RCTs) were included.

#### Exclusion criteria

Studies were excluded if they met any of the following criteria.

(1) Conference abstracts, case reports, study protocols, or systematic reviews.
(2) Studies without sufficient data for analysis.
(3) Studies for which full-text articles were unavailable through relevant databases or other sources.

#### Data extraction

Data were independently extracted for each study that met the inclusion criteria using a predefined data extraction form. Extracted information included study characteristics (first author, publication year, country), participant characteristics (age, sex, sample size), intervention details (exercise type, duration, frequency, and intensity), and outcome measures. For missing data, the corresponding authors were contacted up to three times over three weeks to obtain the required information. Two independent reviewers (GS, BW) performed data extraction, and a third reviewer (LX) cross-checked and adjudicated discrepancies.

#### Data synthesis and analysis

To ensure consistency in the subsequent data synthesis process, this study standardized data encoding, focusing on two key aspects: (1) the overall level, which compares exercise interventions with usual care, and (2) the intervention-specific level, which compares different types of exercise interventions with usual care. At the second level, the interventions were categorized into AT, RT, CT, HIIT, and MBT. Additionally, baseline BMI was modeled both as a continuous predictor and as a categorical variable. Given that 79.5% of the included studies were conducted in Asian populations, with 66.6% specifically in China, we adopted the Chinese BMI classification (WS/T 428-2013),^62^ which was based on large-scale epidemiological evidence from Chinese adults suggesting lower cut-off points for overweight and obesity (24.0 and 28.0 kg/m^2^, respectively) compared with the WHO/ADA criteria.^63^ According to this classification, BMI was categorized as normal weight (18.5–23.9 kg/m^2^), overweight (24.0–27.9 kg/m^2^), and obesity (≥28.0 kg/m^2^).

To quantify the intensity of each exercise modality, the exercise dose was calculated using the formula: Metabolic Equivalent of Task (MET) × exercise duration per session × weekly frequency, expressed as METs·min/week. Exercise intensity classifications were determined according to the 2024 Adult Compendium of Physical Activities (www.pacompendium.com), which provides 1,114 activity-specific MET codes and incorporates newly recognized exercise modalities, such as HIIT, offering an up-to-date and comprehensive framework for estimating energy expenditure across diverse activities.^64^ For CT protocols, which integrate AT and RT within the same session, the weekly METs were derived by summing the energy expenditure of AT and RT components, weighted by their respective durations and MET values. Exercise frequency was defined as the total number of weekly exercise sessions, including multiple sessions performed within a single day. For interventions in which exercise duration, frequency, or prescribed intensity progressively increased, the weekly METs were calculated using the average values of these parameters across the entire intervention period. To ensure accurate estimation of energy expenditure, each exercise session was divided into three phases: warm-up, primary intervention, and cool-down, with the warm-up and cool-down phases quantified independently and included in the total weekly METs. To further enhance the clinical interpretability of our findings, we referred to the 2024 Adult Compendium of Physical Activities. Based on this compendium, the recommended weekly durations of physical activity (minutes per week) were derived using two complementary approaches: first, by applying the updated energy expenditure classifications that define light, moderate, and vigorous intensities as 1.6–2.9 METs, 3.0–5.9 METs, and ≥6.0 METs, respectively; and second, by converting the estimated energy expenditure of each exercise modality into equivalent weekly minutes using the activity-specific MET codes provided in the compendium.

#### Measures of treatment effect

In this meta-analysis, we used mean difference (MD) and standard deviation (SD) changes to evaluate the effects of exercise interventions. If the original study did not directly report SD values, they were estimated based on standard error (SE), 95% confidence interval (CI), p-value, or t-statistics.^65^ For studies that did not report pre-post change SDs, the following formula was used for estimation:

$${SD}_{change}=\sqrt{{SD}_{baseline}^{2}+{SD}_{Post}^{2}-2\times r\times {SD}_{baseline}\times {SD}_{post}}$$

In this formula, the standard deviation of the pre-post difference was calculated, assuming a correlation coefficient of 0.5. This assumption is based on widely accepted literature recognizing a moderate level of measurement reliability. The choice of this value aims to balance potential variability between pre- and post-measurements, ensuring the robustness and reliability of the results.^65^

#### Quality assessment of evidence

This study conducted a comprehensive risk of bias assessment for the included trials using the Cochrane Risk of Bias Tool for Randomized Controlled Trials (ROB 2.0), covering key domains such as random sequence generation, allocation concealment, blinding, missing outcome data, and selective outcome reporting.^66^ The overall risk-of-bias judgement was derived according to the official guidance of the Cochrane Handbook for Systematic Reviews of Interventions (Chapter 8, version 6.5, 2024), which specifies that the overall judgement reflects the least favourable domain-level assessment.^67^ In practice, studies with all domains rated as low risk were classified as overall low risk of bias; if any domain was rated as high risk, or if multiple domains with “some concerns” substantially reduced confidence in the results, the study was judged as high risk; and in all other cases, studies were categorised as having some concerns. Two reviewers independently performed the assessment, and any discrepancies were resolved by discussion and consensus.^66^^,^^67^

We generated funnel plots and performed direct comparison analyses to detect small-study effects and publication bias. Additionally, to systematically evaluate the certainty of the evidence, we applied the CINeMA framework, assessing six key domains: within-study bias, reporting bias, indirectness, imprecision, heterogeneity, and incoherence.^68^^,^^69^ These domains respectively evaluate potential systematic errors within individual studies, the impact of selective reporting and publication bias, the relevance of evidence to the research question, the uncertainty range of effect estimates, the consistency of findings across studies, and the agreement between direct and indirect evidence.

#### Minimally contextualised framework

This study adopted a minimally contextualised framework to conduct the network meta-analysis, aiming to evaluate the relative effectiveness and clinical relevance of each intervention in individuals with prediabetes. The MCID was used as the decision threshold, with usual care set as the reference group. Based on previous literature, a reduction of ≥0.2% in HbA1c from baseline was defined as the threshold for clinically meaningful improvement in this population.^70^ Interventions were categorised into three groups: most effective, intermediate, and least effective, according to whether they met the MCID threshold and the certainty of evidence assessed using the CINeMA framework, which was further classified as high/moderate or low/very low certainty.

### Quantification and statistical analysis

#### Pairwise Meta-Analyses

When at least two studies evaluated the same exercise intervention, we conducted pairwise meta-analyses using a random-effects model. The effect sizes were pooled using MD, and the corresponding 95% CI were calculated.^71^

#### Network meta-analyses

A Bayesian NMA was performed using the multinma package in R (version 4.3.3) to systematically assess the effects of different exercise interventions on HbA1c in individuals with prediabetes. The multinma package, based on a Bayesian statistical framework, allows for simultaneous comparisons of multiple treatment options, providing more robust effect estimates. A network plot was generated to visualize the network structure of different exercise modalities, where nodes represent different interventions and edges indicate direct comparisons between interventions. Treatment effects were estimated using the Markov Chain Monte Carlo method, and a random-effects model was applied to account for heterogeneity across studies.^72^^,^^73^ To ensure comparability of results, MD was selected as the primary effect measure, and 95% Credible Interval (CrI) was calculated. Between-study heterogeneity was assessed using τ^2^, categorized as follows: low (<0.04), low-moderate (0.04–0.16), moderate-high (0.16–0.36), and high (>0.36).^74^^,^^75^

To assess global inconsistency in the network, we compared the consistency model with the unrelated mean effects model, evaluating model fit using residual deviance, Deviance Information Criterion (DIC), and model complexity index (pD).^76^ Local inconsistency was examined using the node-splitting method, which compares direct and indirect evidence. If p < 0.05, significant inconsistency was considered present^76^ (Appendix 5). The transitivity assumption was evaluated by examining baseline characteristics and intervention features across studies to ensure comparability of study populations and intervention conditions, thereby confirming the robustness of the results. To further compare the efficacy of different interventions, we ranked exercise interventions using Surface Under the Cumulative Ranking (SUCRA) scores, which quantify the cumulative ranking probability of each intervention across all comparisons, identifying the most effective exercise modality.^77^ Additionally, 95% prediction intervals were calculated to estimate the potential variability of future study outcomes, providing insights into the expected effects of exercise interventions in real-world settings.^78^ A meta-regression analysis was conducted to explore potential moderating factors influencing treatment effects, examining variables such as age, sex, sample size, exercise frequency, period, session duration, baseline HbA1c, and baseline BMI. To further evaluate the robustness of the findings, three sensitivity analyses were performed: (1) reanalyzing data after adjusting all potential sources of heterogeneity to their median values, (2) varying the assumed pre–post correlations (r = 0.3, 0.5, and 0.7), and (3) excluding studies at high risk of bias. Furthermore, a funnel plot was generated using the netmeta package to assess publication bias, with Egger’s test performed to detect potential asymmetry indicative of publication bias. Finally, adherence data were extracted from each study based on the number of participants completing the intervention and those assigned to the intervention group. Under the Bayesian framework, adherence was modelled on the log(OR) scale and reported as OR with corresponding 95% CrI.

#### Dose-response meta-analysis

To investigate the dose-response relationship between different exercise modalities and HbA1c (%) in individuals with prediabetes, a Bayesian random-effects dose-response meta-analysis was conducted using the brms package (version 2.18.0) in R.^79^ Data preprocessing and visualization were performed using the tidybayes package^80^ (version 3.0.2). At the same time, the graphical representation was completed with ggplot2^81^ (version 3.3.6). The modeling process used a normal likelihood function with an identity link function to model changes in HbA1c while adjusting for weekly exercise dose and baseline BMI levels. Expressly, both linear and nonlinear terms (e.g., natural splines) were incorporated into the model, following Harrell’s modeling strategy.^82^ We evaluated several fit indices to select the most appropriate model, including log-pointwise predictive density, standard error, the number of effective parameters, and leave-one-out cross-validation information criterion. The final model employed natural splines with four knots to model the dose-response relationship between physical activity dose and baseline BMI levels, achieving the best model fit. To explore the independent effect of exercise intensity, we applied the same Bayesian spline modeling framework as used in the total exercise dose analysis. The model simultaneously included weekly exercise duration (minutes) and exercise intensity (METs) to estimate their independent effects. For ease of interpretation, we fixed the duration at the sample median (150 minutes/week) during the prediction stage, thereby presenting the dose–response relationship between intensity and the outcome at a given duration.

Based on the best-fit model, we estimated the optimal exercise dose for maximum HbA1c reduction across different BMI categories defined by the Chinese classification (WS/T 428-2013) (normal weight, overweight, obesity).^62^^,^^63^ Additionally, we calculated the minimum effective dose required to achieve a clinically significant change in HbA1c (%). In this study, an HbA1c reduction of ≥ -0.2% from baseline was considered clinically meaningful.^70^

### Additional resources

The protocol for this systematic review and network meta-analysis has been registered in PROSPERO (ID: CRD420250653913). This study follows the PRISMA 2020 (Preferred Reporting Items for Systematic Reviews and Meta-Analyses) guidelines and the extended PRISMA-NMA statement for network meta-analysis.^83^^,^^84^

All data in this study were collected from the included literature and do not require ethical approval.

## Supplemental information

Document S1. Table S1–Table S11 and Figure S4–S11

Methods S1. Definitions of Exercise Interventions

Table S12. Baseline of characteristics of included studies

Table S13. Minimal effective and optimal exercise doses for each exercise modality by single-unit BMI categories
