# Supplementary material for: Optimal exercise dose for glycemic control in prediabetes across different exercise types
Source: iScience. 2025 Nov 10;28(12):113980. doi: 10.1016/j.isci.2025.113980 (PMC12689185; doi:10.1016/j.isci.2025.113980)
Supplement: Document S1. Table S1–Table S11 and Figure S4–S11 [file mmc1.pdf]

iScience, Volume *28*

Supplemental information

Optimal exercise dose for glycemic control in prediabetes across different exercise types

Ruixiang Yan, Yueming Li, Shiqi Jia, Jiaxin He, Gesheng Lin, Weifeng Huang, Jian Sun, and Duanying Li

Supplementary Appendix

Table of contents

Appendix 1: Search strategy

Appendix 2: Risk of bias of randomized clinical trials

Appendix 3: Evaluation of inconsistency and heterogeneity

Appendix 4: Forest plots of Network Meta-Analysis

Appendix 5: SUCRA and cumulative probability plots

Appendix 6: Forest Plot of Pairwise Comparisons from Meta-Analysis

Appendix 7: Dose-response relationship between exercise dose and HbA1c reduction.

Appendix 8: CINeMA Assessment

Appendix 9: Funnel plots

Appendix10: Network Meta-Regression

Appendix11: Sensitivity analysis

Appendix 1: Search strategy

Table S1.1: Search strategy of Pubmed

#	Searches
1	((((((((((((((((Exercises[MeSH Terms]) OR (Exercise,Physical[Title/Abstract])) OR (Physical Exercise[Title/Abstract])) OR (Physical Exercises[Title/Abstract])) OR (Exercises, Physical[Title/Abstract])) OR (Physical Activity[Title/Abstract])) OR (Activities, Physical[Title/Abstract])) OR (Activity, Physical[Title/Abstract])) OR (Physical Activities[Title/Abstract])) OR (Exercise, Aerobic[Title/Abstract])) OR (Aerobic Exercise[Title/Abstract])) OR (Aerobic Exercises[Title/Abstract])) OR (Exercises, Aerobic[Title/Abstract])) OR (Exercise Training[Title/Abstract])) OR (Exercise Trainings[Title/Abstract])) OR (Training, Exercise[Title/Abstract])) OR (Trainings, Exercise[Title/Abstract])
2	(((((((((((((((((((((((Training, Resistance[Title/Abstract]) OR (Strength Training[Title/Abstract])) OR (Training, Strength[Title/Abstract])) OR (Weight-Lifting Strengthening Program[Title/Abstract])) OR (Strengthening Programs, Weight-Lifting[Title/Abstract])) OR (Strengthening Program, Weight-Lifting[Title/Abstract])) OR (Weight Lifting Strengthening Program[Title/Abstract])) OR (Weight-Lifting Strengthening Programs[Title/Abstract])) OR (Weight-Lifting Exercise Program[Title/Abstract])) OR (Exercise Programs, Weight-Lifting[Title/Abstract])) OR (Exercise Program, Weight-Lifting[Title/Abstract])) OR (Weight Lifting Exercise Program[Title/Abstract])) OR (Weight-Lifting Exercise Programs[Title/Abstract])) OR (Weight-Bearing Strengthening Program[Title/Abstract])) OR (Strengthening Programs, Weight-Bearing[Title/Abstract])) OR (Strengthening Program, Weight-Bearing[Title/Abstract])) OR (Weight Bearing Strengthening Program[Title/Abstract])) OR (Weight-Bearing Strengthening Programs[Title/Abstract])) OR (Weight-Bearing Exercise Program[Title/Abstract])) OR (Exercise Programs, Weight-Bearing[Title/Abstract])) OR (Exercise Program, Weight-Bearing[Title/Abstract])) OR (Weight Bearing Exercise Program[Title/Abstract])) OR (Weight-Bearing Exercise Programs[Title/Abstract])) OR (Resistance training[MeSH Terms])
3	((((((((Cardiovascular Exercise[Title/Abstract]) OR (Endurance Training[Title/Abstract])) OR (Walking[Title/Abstract])) OR (Running[Title/Abstract])) OR (Cycling[Title/Abstract])) OR (Swimming[Title/Abstract])) OR (Dancing[Title/Abstract])) OR (combined training[Title/Abstract])) OR (Concurrent Training[Title/Abstract])
4	((((((((((((High Intensity Interval Training[Title/Abstract]) OR (High-Intensity Interval Trainings[Title/Abstract])) OR (Interval Training, High-Intensity[Title/Abstract])) OR (Interval Trainings, High-Intensity[Title/Abstract])) OR (Training, High-Intensity Interval[Title/Abstract])) OR (Trainings, High-Intensity Interval[Title/Abstract])) OR (High-Intensity Intermittent Exercise[Title/Abstract])) OR (Exercise, High-Intensity Intermittent[Title/Abstract])) OR (Exercises, High-Intensity Intermittent[Title/Abstract])) OR (High-Intensity Intermittent Exercises[Title/Abstract])) OR (Sprint Interval Training[Title/Abstract])) OR (Sprint Interval Trainings[Title/Abstract])) OR (High-Intensity Interval Training[MeSH Terms])
5	((((((Circuit-Based Exercise[MeSH Terms]) OR (Circuit Based Exercise[Title/Abstract])) OR (Circuit-Based Exercises[Title/Abstract])) OR (Exercise, Circuit-Based[Title/Abstract])) OR (Exercises, Circuit-Based[Title/Abstract])) OR (Circuit Training[Title/Abstract])) OR (Training, Circuit[Title/Abstract])
6	(((((((((((((((((Chinese exercise[Title/Abstract]) OR (traditional exercise[Title/Abstract])) OR (traditional chinese medicine[Title/Abstract])) OR (chinese traditional exercise[Title/Abstract])) OR (traditional chinese exercise[Title/Abstract])) OR (traditional exercise[Title/Abstract])) OR (traditional Kungfu[Title/Abstract])) OR (Kungfu[Title/Abstract])) OR (Shadowboxing[Title/Abstract])) OR (Five-animal exercises[Title/Abstract])) OR

	(Wuqinxi[Title/Abstract])) OR (Five animal frolics[Title/Abstract])) OR (Five-animal boxing[Title/Abstract])) OR (Baduanjin[Title/Abstract])) OR (Yijinjing[Title/Abstract])) OR (liuzijue[Title/Abstract])) OR (((Qigong[MeSH Terms]) OR (Ch'i Kung[Title/Abstract])) OR (Qi Gong[Title/Abstract]))) OR (((((((((taiji[MeSH Terms]) OR (Tai-ji[Title/Abstract])) OR (Tai Chi[Title/Abstract])) OR (Chi, Tai[Title/Abstract])) OR (Tai Chi Chuan[Title/Abstract])) OR (Taijiquan[Title/Abstract])) OR (T'ai Chi[Title/Abstract])) OR (Tai Ji Quan[Title/Abstract])) OR (Ji Quan, Tai[Title/Abstract])) OR (Quan, Tai Ji[Title/Abstract]))
7	(((Mind-body exercises[Title/Abstract]) OR (yoga[Title/Abstract])) OR (dance[Title/Abstract])) OR (Pilates[Title/Abstract])
8	((((((((((((Prediabetic State[MeSH Terms]) OR (Prediabetic States[Title/Abstract])) OR (State, Prediabetic[Title/Abstract])) OR (States, Prediabetic[Title/Abstract])) OR (Prediabetes[Title/Abstract])) OR (Pre-diabetes[Title/Abstract])) OR (impaired glucose[Title/Abstract])) OR (impaired fasting glucose[Title/Abstract])) OR (impaired glucose intolerance[Title/Abstract])) OR (impaired glucose tolerance[Title/Abstract])) OR (impaired glucose regulation[Title/Abstract])) OR (borderline diabetes[Title/Abstract])) OR (higher risk of diabetes[Title/Abstract])) OR (high risk of diabetes[Title/Abstract])
9	(((((randomized controlled trial[Publication Type]) OR (randomized)) OR (clinical trials as topic[MeSH Terms])) OR (placebo)) OR (randomly)) OR (trial)
10	#1 OR #2 OR #3 OR #4 OR #5 OR #6 OR #7
11	#8 AND #9 AND #10

Table S1.2: Search strategy of Web of Science

#	Searches
1	TS=("Prediabetic State" OR "Prediabetic States" OR "States, Prediabetic" OR "Prediabetes" OR "impaired glucose" OR "impaired fasting glucose" OR "impaired glucose intolerance" OR "impaired glucose tolerance" OR "impaired glucose regulation" OR "borderline diabetes" OR "higher risk of diabetes" OR "high risk of diabetes")
2	TS=("Exercise" OR "Exercises" OR "Exercise, Physical" OR "Exercises, Physical" OR "Physical Exercise" OR "Physical Exercises" OR "Physical Activity" OR "Activities, Physical" OR "Activity, Physical" OR "Physical Activities" OR "Exercise, Aerobic" OR "Aerobic Exercise" OR "Aerobic Exercises" OR "Exercises, Aerobic" OR "Exercise Training" OR "Exercise Trainings" OR "Training, Exercise" OR "Trainings, Exercise")
3	TS=("Resistance training" OR "Training, Resistance" OR "Strength Training" OR "Training, Strength" OR "Weight-Lifting Strengthening Program" OR "Strengthening Programs, Weight-Lifting" OR "Strengthening Program, Weight-Lifting" OR "Weight Lifting Strengthening Program" OR "Weight-Lifting Strengthening Programs" OR "Weight-Lifting Exercise Program" OR "Exercise Programs, Weight-Lifting" OR "Exercise Program, Weight-Lifting" OR "Weight Lifting Exercise Program" OR "Weight-Lifting Exercise Programs" OR "Weight-Bearing Strengthening Program" OR "Strengthening Programs, Weight-Bearing" OR "Strengthening Program, Weight-Bearing" OR "Weight Bearing Strengthening Program" OR "Weight-Bearing Strengthening Programs" OR "Weight-Bearing Exercise Program" OR "Exercise Programs, Weight-Bearing" OR "Exercise Program, Weight-Bearing" OR "Weight Bearing Exercise Program" OR "Weight-Bearing Exercise Programs")
4	TS=("Cardiovascular Exercise" OR "Endurance Training" OR "Walking" OR "Running" OR "Cycling" OR "Swimming" OR "Dancing" OR "combined training" OR "Concurrent Training")
5	TS=("High-Intensity Interval Training" OR "High Intensity Interval Training" OR "High-Intensity Interval Trainings" OR "Interval Training, High-Intensity" OR "Interval Trainings, High-Intensity" OR "Training, High-Intensity Interval" OR "Trainings, High-Intensity Interval" OR "High-Intensity Intermittent Exercise" OR "Exercise, High-Intensity Intermittent" OR "Exercises, High-Intensity Intermittent" OR "High-Intensity Intermittent Exercises" OR "Sprint Interval Training" OR "Sprint Interval Trainings")
6	TS=("Circuit-Based Exercise" OR "Circuit Based Exercise" OR "Circuit-Based Exercises" OR "Exercise, Circuit-Based" OR "Exercises, Circuit-Based" OR "Circuit Training" OR "Training, Circuit")
7	TS=("Chinese exercise" OR "traditional exercise" OR "traditional chinese medicine" OR "chinese traditional exercise" OR "traditional chinese exercise" OR "traditional Kungfu" OR "Kungfu" OR "Shadowboxing" OR "Five-animal exercises" OR "Wuqinxi" OR "Five animal frolics" OR "Five-animal boxing" OR "Baduanjin" OR "Yijinjing" OR "liuzijue" OR "Qigong" OR "Ch'i Kung" OR "Qi Gong" OR "taiji" OR "Tai-ji" OR "Tai Chi" OR "Chi, Tai" OR "Tai Chi Chuan" OR "Taijiquan" OR "T'ai Chi" OR "Tai Ji Quan" OR "Ji Quan, Tai" OR "Quan, Tai Ji")
8	TS=("Mind-body exercises" OR "yoga" OR "dance" OR "Pilates")
9	TS=("randomized controlled trial" OR "randomized" OR "clinical trials" OR "placebo" OR "randomly" OR "trial")
10	#2 OR #3 OR #4 OR #5 OR #6 OR #7 OR #8
11	#1 AND #9 AND #10

Table S1.3: Search strategy of Cochrane Central Register of Controlled Trials

#	Searches
1	MeSH descriptor: [Prediabetic State] explode all trees
2	MeSH descriptor: [High-Intensity Interval Training] explode all trees
3	MeSH descriptor: [Exercise] explode all trees
4	MeSH descriptor: [Circuit-Based Exercise] explode all trees
5	MeSH descriptor: [Resistance Training] explode all trees
6	MeSH descriptor: [Tai Ji] explode all trees
7	MeSH descriptor: [Qigong] explode all trees
8	MeSH descriptor: [Yoga] explode all trees
9	MeSH descriptor: [Exercise Movement Techniques] explode all trees
10	("aerobic exercise":ti,ab,kw OR "aerobic training":ti,ab,kw OR "Cardiovascular Exercise":ti,ab,kw OR "Endurance Training":ti,ab,kw OR "Walking":ti,ab,kw OR "Running":ti,ab,kw OR "Cycling":ti,ab,kw OR "Swimming":ti,ab,kw OR "Dancing":ti,ab,kw OR "combined training":ti,ab,kw OR "Concurrent Training":ti,ab,kw OR "Mind-body exercises":ti,ab,kw OR "yoga":ti,ab,kw OR "dance":ti,ab,kw OR "pilates":ti,ab,kw OR "traditional chinese medicine":ti,ab,kw OR "chinese traditional exercise":ti,ab,kw OR "traditional exercise":ti,ab,kw OR "traditional Kungfu":ti,ab,kw OR "Kungfu":ti,ab,kw OR "Shadowboxing":ti,ab,kw OR "Wuqinxi":ti,ab,kw OR "Five-animal exercises":ti,ab,kw OR "Five animal frolics":ti,ab,kw OR "Five-animal boxing":ti,ab,kw OR "Baduanjin":ti,ab,kw OR "Yijinjing":ti,ab,kw OR "liuzijue":ti,ab,kw)
11	#2 OR #3 OR #4 OR #5 OR #6 OR #7 OR #8 OR #9 OR #10
12	#1 AND #11

Table S1.4: Search strategy of Embase

#	Searches
1	'Prediabetic State'/exp OR 'Prediabetic State':ab,ti OR 'Prediabetic States':ab,ti OR 'States, Prediabetic':ab,ti OR 'Prediabetes':ab,ti OR 'impaired glucose':ab,ti OR 'impaired fasting glucose':ab,ti OR 'impaired glucose intolerance':ab,ti OR 'impaired glucose tolerance':ab,ti OR 'impaired glucose regulation':ab,ti OR 'borderline diabetes':ab,ti OR 'higher risk of diabetes':ab,ti OR 'high risk of diabetes':ab,ti
2	'Exercise'/exp OR 'Exercise':ab,ti OR 'Exercises':ab,ti OR 'Exercise, Physical':ab,ti OR 'Exercises, Physical':ab,ti OR 'Physical Exercise':ab,ti OR 'Physical Exercises':ab,ti OR 'Physical Activity':ab,ti OR 'Activities, Physical':ab,ti OR 'Activity, Physical':ab,ti OR 'Physical Activities':ab,ti OR 'Exercise, Aerobic':ab,ti OR 'Aerobic Exercise':ab,ti OR 'Aerobic Exercises':ab,ti OR 'Exercises, Aerobic':ab,ti OR 'Exercise Training':ab,ti OR 'Exercise Trainings':ab,ti OR 'Training, Exercise':ab,ti OR 'Trainings, Exercise':ab,ti
3	'Resistance training'/exp OR 'Resistance training':ab,ti OR 'Training, Resistance':ab,ti OR 'Strength Training':ab,ti OR 'Training, Strength':ab,ti OR 'Weight-Lifting Strengthening Program':ab,ti OR 'Strengthening Programs, Weight-Lifting':ab,ti OR 'Strengthening Program, Weight-Lifting':ab,ti OR 'Weight Lifting Strengthening Program':ab,ti OR 'Weight-Lifting Strengthening Programs':ab,ti OR 'Weight-Lifting Exercise Program':ab,ti OR 'Exercise Programs, Weight-Lifting':ab,ti OR 'Exercise Program, Weight-Lifting':ab,ti OR 'Weight Lifting Exercise Program':ab,ti OR 'Weight-Lifting Exercise Programs':ab,ti OR 'Weight-Bearing Strengthening Program':ab,ti OR 'Strengthening Programs, Weight-Bearing':ab,ti OR 'Strengthening Program, Weight-Bearing':ab,ti OR 'Weight Bearing Strengthening Program':ab,ti OR 'Weight-Bearing Strengthening Programs':ab,ti OR 'Weight-Bearing Exercise Program':ab,ti OR 'Exercise Programs, Weight-Bearing':ab,ti OR 'Exercise Program, Weight-Bearing':ab,ti OR 'Weight Bearing Exercise Program':ab,ti OR 'Weight-Bearing Exercise Programs':ab,ti
4	'Cardiovascular Exercise':ab,ti OR 'Endurance Training':ab,ti OR 'Walking':ab,ti OR 'Running':ab,ti OR 'Cycling':ab,ti OR 'Swimming':ab,ti OR 'Dancing':ab,ti OR 'combined training':ab,ti OR 'Concurrent Training':ab,ti
5	'High-Intensity Interval Training'/exp OR 'High Intensity Interval Training':ab,ti OR 'High-Intensity Interval Trainings':ab,ti OR 'Interval Training, High-Intensity':ab,ti OR 'Interval Trainings, High-Intensity':ab,ti OR 'Training, High-Intensity Interval':ab,ti OR 'Trainings, High-Intensity Interval':ab,ti OR 'High-Intensity Intermittent Exercise':ab,ti OR 'Exercise, High-Intensity Intermittent':ab,ti OR 'Exercises, High-Intensity Intermittent':ab,ti OR 'High-Intensity Intermittent Exercises':ab,ti OR 'Sprint Interval Training':ab,ti OR 'Sprint Interval Trainings':ab,ti
6	'Circuit-Based Exercise'/exp OR 'Circuit Based Exercise':ab,ti OR 'Circuit-Based Exercises':ab,ti OR 'Exercise, Circuit-Based':ab,ti OR 'Exercises, Circuit-Based':ab,ti OR 'Circuit Training'/exp OR 'Training, Circuit':ab,ti
7	'chinese exercise':ab,ti OR 'traditional exercise':ab,ti OR 'traditional chinese medicine':ab,ti OR 'chinese traditional exercise':ab,ti OR 'traditional chinese exercise':ab,ti OR 'traditional kungfu':ab,ti OR 'kungfu':ab,ti OR 'shadowboxing':ab,ti OR 'five-animal exercises':ab,ti OR 'wuqinxi':ab,ti OR 'five animal frolics':ab,ti OR 'five-animal boxing':ab,ti OR 'baduanjin':ab,ti OR 'yijinjing':ab,ti OR 'liuzijue':ab,ti OR 'qigong'/exp OR 'chi kung':ab,ti OR 'qi gong':ab,ti OR 'taiji' OR 'tai-ji':ab,ti OR 'chi, tai':ab,ti OR 'tai chi chuan':ab,ti OR 'taijiquan':ab,ti OR 'tai chi':ab,ti OR 'tai ji quan':ab,ti OR 'ji quan, tai':ab,ti OR 'quan, tai ji':ab,ti
8	'Mind-body exercises':ab,ti OR 'yoga'/exp OR 'dance':ab,ti OR 'Pilates'/exp
9	'randomized controlled trial'/exp OR 'randomized':ab,ti OR 'clinical trials':ab,ti OR 'placebo':ab,ti OR 'randomly':ab,ti OR 'trial':ab,ti
10	#2 OR #3 OR #4 OR #5 OR #6 OR #7 OR #8

11 #1 AND #9 AND #10

Table S1.5: Search strategy of CNKI

#	Searches
1	TKA%=(中医运动+传统功法+中国传统运动+五禽戏+太极+太极拳+八段锦+易筋经+功夫+气功+六字诀+身心运动+瑜伽+舞蹈+普拉提+抗阻训练＋阻力训练＋抗阻运动+力量训练+有氧运动+耐力训练+心肺锻炼+跳绳+有氧训练+跑步+游泳+长跑+骑行+广场舞+健身操+慢跑+自行车+步行+健步走+联合运动+同期训练+循环训练) *(糖尿病前期+空腹血糖受损+糖耐量受损+血糖受损调节＋糖调节受损)

Table S1.6: Search strategy of WanFang Data

#	Searches
1	主题:(中医运动 OR 传统功法 OR 中国传统运动 OR 五禽戏 OR 太极 OR 太极拳 OR 八段锦 OR 易筋经 OR 功夫 OR 气功 OR 六字诀 OR 身心运动 OR 瑜伽 OR 舞蹈 OR 普拉提 OR 抗阻训练 OR 阻力训练 OR 抗阻运动 OR 力量训练 OR 有氧运动 OR 耐力训练 OR 心肺锻炼 OR 跳绳 OR 有氧训练 OR 跑步 OR 游泳 OR 长跑 OR 骑行 OR 广场舞 OR 健身操 OR 慢跑 OR 自行车 OR 步行 OR 健步走 OR 联合运动 OR 同期训练 OR 循环训练)
	主题:(糖尿病前期 OR 空腹血糖受损 OR 糖耐量受损 OR 血糖受损调节 OR 糖调节受损)
	#1 AND #2

Appendix 2: Risk of bias of randomized clinical trials

Table S2.1: Study level risk of bias assessment using Cochrane risk of bias tool 2.0 for assessing risk of bias of randomized clinical trials.

Unique ID	Study ID	Randomization process	Deviations from intended interventions	Missing outcome data	Measurement of the outcome	Selection of the reported result	Overall Bias
Badaam et al.2021	1	Low	Low	Low	Low	Low	Low
Cai et al.2023	2	Low	Low	Low	Low	Some concerns	Some concerns
Chen et al.2021	3	Low	Low	Low	Low	Some concerns	Some concerns
Cheng et al.2017	4	Low	Low	Low	Low	Low	Low
Dai et al.2015	5	Some concerns	Low	Low	Low	Some concerns	Some concerns
Desch et al.2010	6	Some concerns	Low	Low	Low	Some concerns	Some concerns
Fritz et al.2013	7	Low	Low	Low	Low	Some concerns	Some concerns
Gidlund et al.2016	8	Some concerns	Low	Low	Low	Some concerns	Some concerns
Gilbertson et al.2019	9	Some concerns	Low	High	Low	Some concerns	High
Hegde et al.2013	10	Some concerns	Low	Low	Low	Some concerns	Some concerns
Hu et al.2022	11	Some concerns	Low	Low	Low	Some concerns	Some concerns
Ji et al.2017	12	Low	Low	Low	Low	Some concerns	Some concerns
Kimi et al.2022	13	Some concerns	Low	High	Low	Some concerns	High
Kramer et al.2018	14	Low	Low	Low	Low	Some concerns	Some concerns
Li et al.2018a	15	Some concerns	Low	Low	Low	Some concerns	Some concerns
Li et al.2018b	16	Some concerns	Low	Low	Low	Some concerns	Some concerns
Li et al.2018c	17	Low	Low	Low	Low	Some concerns	Some concerns
Liao et al.2015	18	Some concerns	Low	Low	Low	Some concerns	Some concerns
Liu et al.2021	19	Low	Low	Low	Low	Low	Low
Luo et al.2017	20	Low	Low	Low	Low	Some concerns	Some concerns
Ma et al.2017	21	Some concerns	Low	High	Low	Some concerns	High
Ma et al.2022	22	Low	Low	High	Low	Some concerns	High
Martins et al.2018	23	Low	Low	Low	Low	Low	Low

RezkAllah et al.2019	24	Low	Low	Low	Low	Low	Low
Rowan et al.2017	25	Low	Low	Low	Low	Some concerns	Some concerns
Ruan et al.2024	26	Some concerns	Low	Low	Low	Some concerns	Some concerns
Safarimosavi et al.2021	27	Low	Low	Low	Low	Some concerns	Some concerns
Venojärvi et al.2013	28	Some concerns	Low	Low	Low	Some concerns	Some concerns
Wang et al.2015	29	Some concerns	Low	Low	Low	Some concerns	Some concerns
Wang et al.2021	30	Some concerns	Low	Low	Low	Some concerns	Some concerns
Wang et al.2023	31	Some concerns	Low	Low	Low	Some concerns	Some concerns
Wei et al.2017	32	Some concerns	Low	Low	Low	Some concerns	Some concerns
Wei et al.2024	33	Some concerns	Low	Low	Low	Some concerns	Some concerns
Wu et al.2016	34	Some concerns	Low	Low	Low	Some concerns	Some concerns
Yan et al.2019	35	Low	Low	Low	Low	Low	Low
Yuan et al.2020	36	Low	Low	Low	Low	Low	Low
Zhang et al.2019	37	Some concerns	Low	Low	Low	Some concerns	Some concerns
Zhang et al.2023	38	Some concerns	Low	High	Low	Some concerns	High
Zheng et al.2017	39	Some concerns	Low	Low	Low	Some concerns	Some concerns

Appendix 3: Evaluation of inconsistency and heterogeneity

Table S3.1: Evaluation of consistency, global inconsistency, and heterogeneity (τ^2) for HbA1c outcomes.

Outcomes	Study	τ^2	Consistency			Global inconsistency		
			Residual deviance	pD	DIC	Residual deviance	pD	DIC
HbA1c	39 studies, 55 RCT, n= 4394	0.016	135.6	100.5	236.1	136.5	100.5	237

Figure S3.1: Density Plots for Node-Splitting Analysis

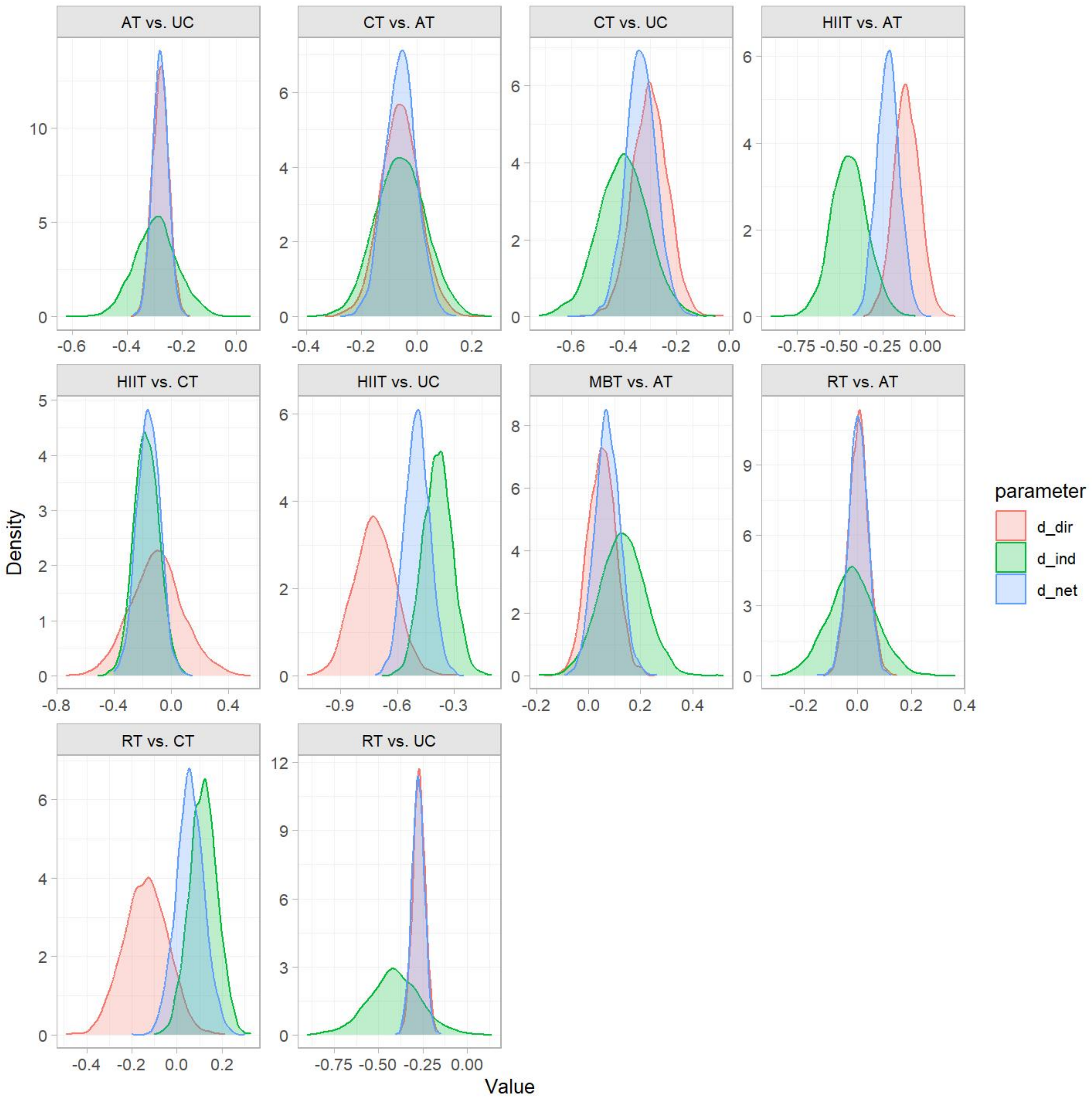

Table S3.2: Side-splitting of HbA1c. Inconsistency test between direct and indirect treatment comparisons with heterogeneity (τ^2) estimates in mixed treatment comparison.

Comparison		mean	sd	2.50%	25%	50%	75%	97.50%	Bayesian p-value	τ^2
AT vs.UC	d_net	-0.28	0.03	-0.33	-0.3	-0.28	-0.26	-0.22	0.82	0.015
	d_dir	-0.28	0.03	-0.34	-0.3	-0.28	-0.26	-0.22		
	d_ind	-0.29	0.08	-0.44	-0.35	-0.29	-0.24	-0.13		
	omega	0.02	0.08	-0.14	-0.04	0.02	0.07	0.17		
CT vs.UC	d_net	-0.34	0.06	-0.45	-0.37	-0.34	-0.3	-0.22	0.37	0.002
	d_dir	-0.3	0.07	-0.43	-0.35	-0.3	-0.26	-0.17		
	d_ind	-0.4	0.1	-0.6	-0.47	-0.4	-0.34	-0.22		
	omega	0.1	0.11	-0.12	0.03	0.1	0.18	0.33		
HIIT vs.UC	d_net	-0.49	0.07	-0.62	-0.54	-0.49	-0.45	-0.36	0.013	0.068
	d_dir	-0.72	0.11	-0.92	-0.79	-0.72	-0.65	-0.5		
	d_ind	-0.39	0.08	-0.53	-0.44	-0.39	-0.33	-0.24		
	omega	-0.33	0.13	-0.59	-0.43	-0.33	-0.24	-0.06		
RT vs.UC	d_net	-0.28	0.04	-0.35	-0.3	-0.28	-0.25	-0.21	0.34	0.029
	d_dir	-0.27	0.04	-0.34	-0.29	-0.27	-0.25	-0.2		
	d_ind	-0.41	0.15	-0.7	-0.51	-0.41	-0.31	-0.11		
	omega	0.14	0.15	-0.16	0.04	0.14	0.24	0.44		
CT vs.AT	d_net	-0.06	0.06	-0.17	-0.1	-0.06	-0.02	0.05	1	0.003
	d_dir	-0.06	0.07	-0.2	-0.11	-0.06	-0.01	0.08		
	d_ind	-0.06	0.09	-0.23	-0.12	-0.06	0	0.12		
	omega	0	0.11	-0.22	-0.08	0	0.08	0.21		
HIIT vs.AT	d_net	-0.22	0.06	-0.34	-0.26	-0.22	-0.17	-0.09	0.015	0.001
	d_dir	-0.11	0.07	-0.26	-0.16	-0.11	-0.06	0.04		
	d_ind	-0.44	0.11	-0.65	-0.51	-0.44	-0.37	-0.23		
	omega	0.33	0.13	0.08	0.25	0.33	0.42	0.59		
MBT vs.AT	d_net	0.07	0.05	-0.02	0.04	0.07	0.11	0.17	0.41	0.004

	**d_dir**	0.05	0.05	-0.05	0.01	0.05	0.09	0.15		
	**d_ind**	0.13	0.09	-0.04	0.07	0.13	0.19	0.3		
	**omega**	-0.08	0.1	-0.27	-0.14	-0.08	-0.01	0.11		
**RT vs.AT**	**d_net**	0	0.04	-0.07	-0.02	0	0.03	0.07	0.78	0.008
	**d_dir**	0	0.04	-0.07	-0.02	0	0.03	0.08		
	**d_ind**	-0.02	0.09	-0.2	-0.08	-0.02	0.04	0.16		
	**omega**	0.02	0.09	-0.16	-0.04	0.03	0.09	0.21		
**HIIT vs.CT**	**d_net**	-0.16	0.08	-0.32	-0.21	-0.16	-0.1	-0.01	0.74	0
	**d_dir**	-0.1	0.18	-0.46	-0.22	-0.1	0.01	0.28		
	**d_ind**	-0.17	0.09	-0.34	-0.23	-0.17	-0.11	0		
	**omega**	0.07	0.2	-0.32	-0.06	0.07	0.2	0.48		
**RT vs.CT**	**d_net**	0.06	0.06	-0.06	0.02	0.06	0.1	0.18	<0.01	0
	**d_dir**	-0.14	0.1	-0.33	-0.2	-0.14	-0.07	0.05		
	**d_ind**	0.12	0.06	0	0.08	0.12	0.16	0.24		
	**omega**	-0.26	0.1	-0.45	-0.32	-0.26	-0.19	-0.06		

Figure S3.2: Network Contribution Heatmap: Evaluation of Direct and Indirect Comparison Contributions in Network Meta-analysis.

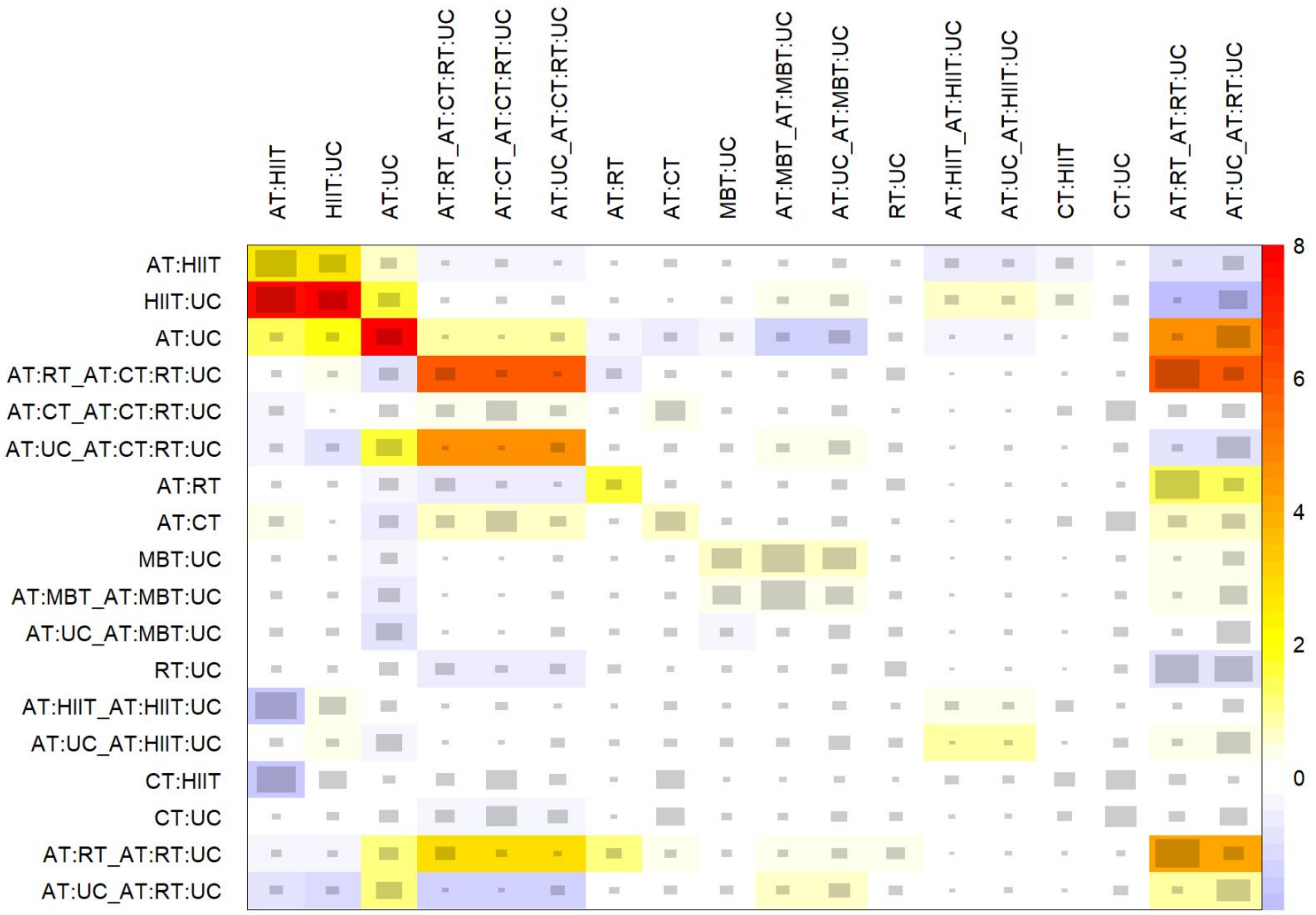

Appendix 4: Forest plots of Network Meta-Analysis

Figure S4.1: Forest Plot of Pairwise Comparisons from Network Meta-Analysis on HbA1c levels

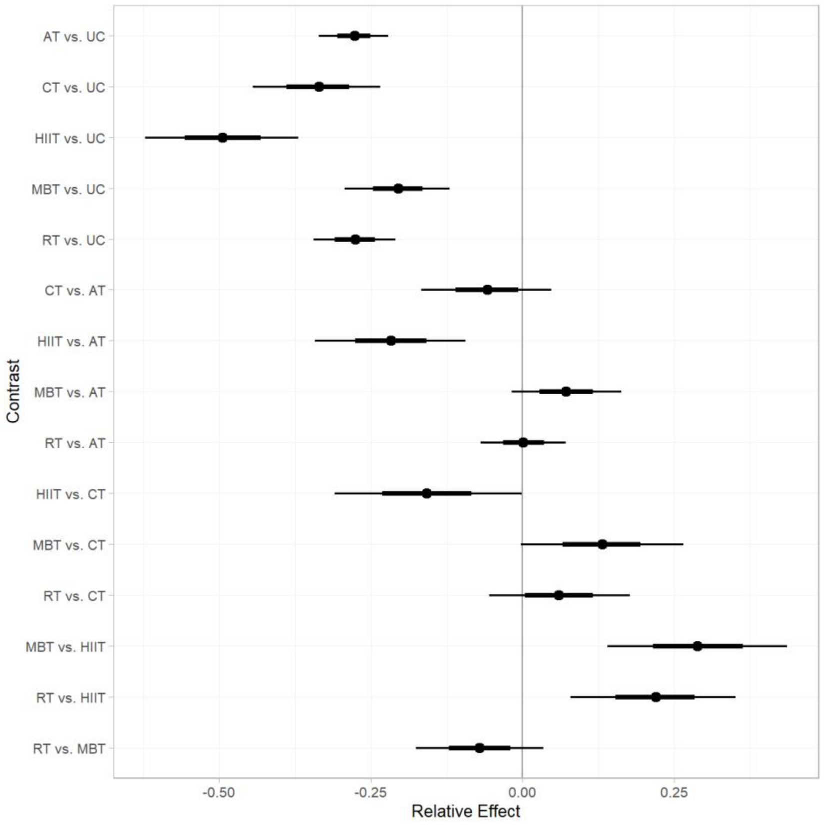

Figure S4.2: Forest Plot of Pairwise Comparisons with Predictive Intervals in Network Meta-Analysis on HbA1c levels

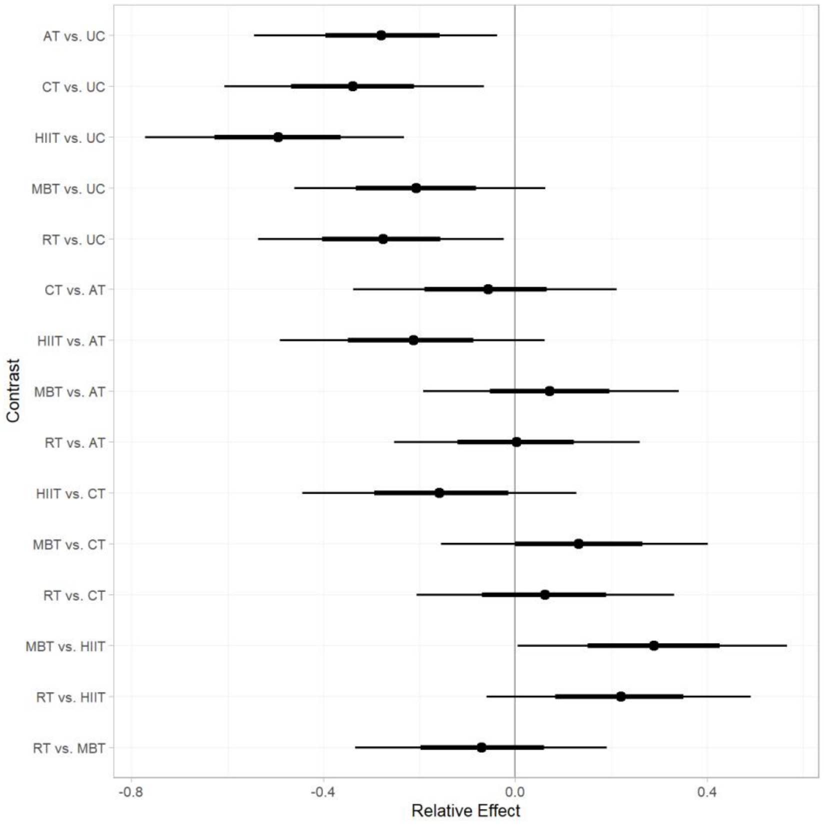

Figure S4.3: Forest Plot of Pairwise Comparisons from Network Meta-Analysis on on Adherence

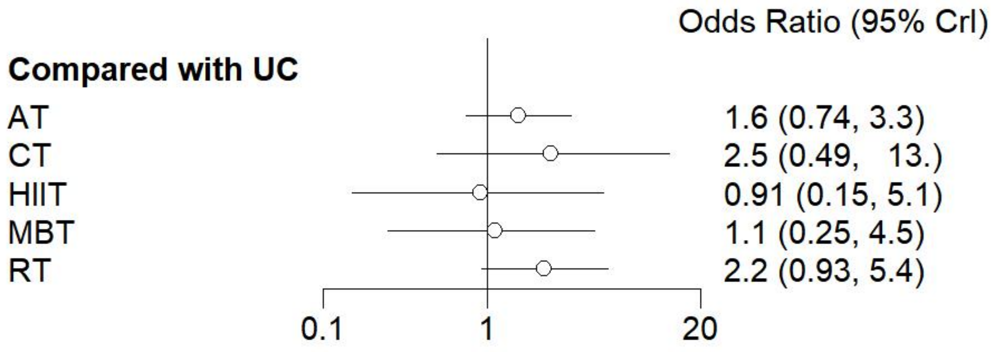

Appendix 5: SUCRA and cumulative probability plots

Figure S5.1: Intervention Ranking Plot for Different Exercise Modalities in HbA1c Network Analysis.

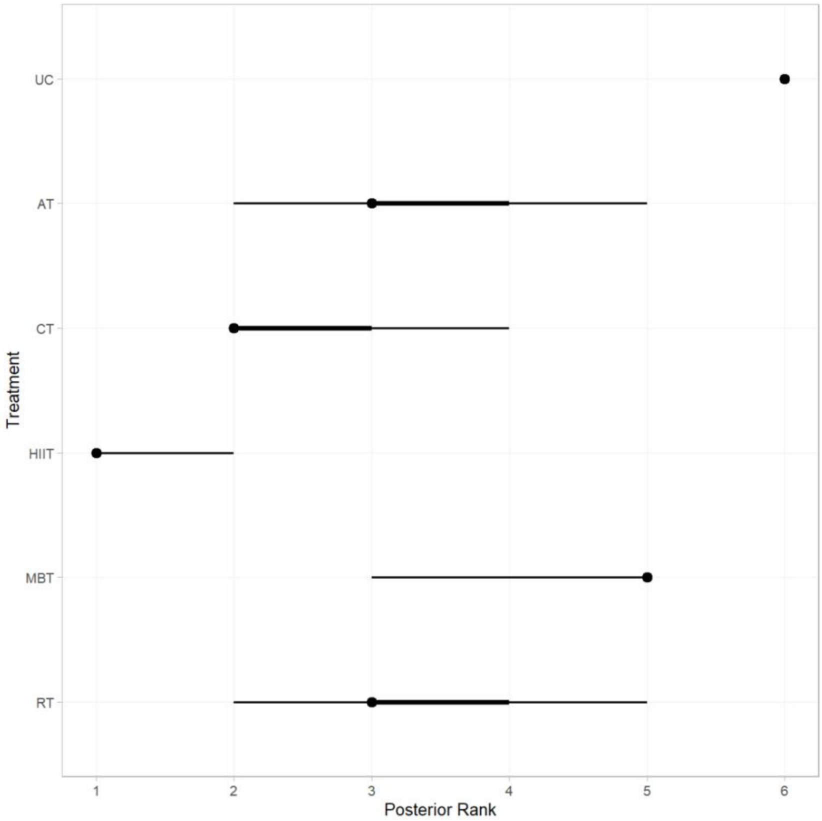

Figure S5.2：Probability of Rank Distribution Plot for Different Exercise Modalities in HbA1c Network Analysis.

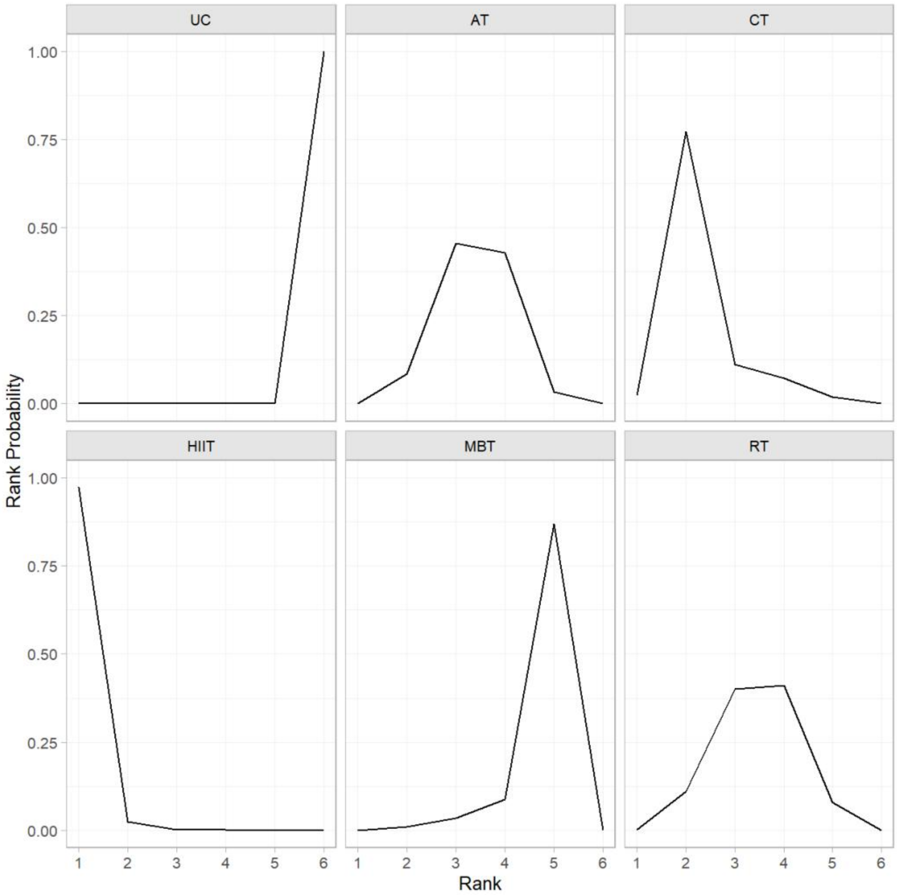

Figure S5.3： Cumulative Probability Plot for Different Exercise Modalities in HbA1c Network Analysis.

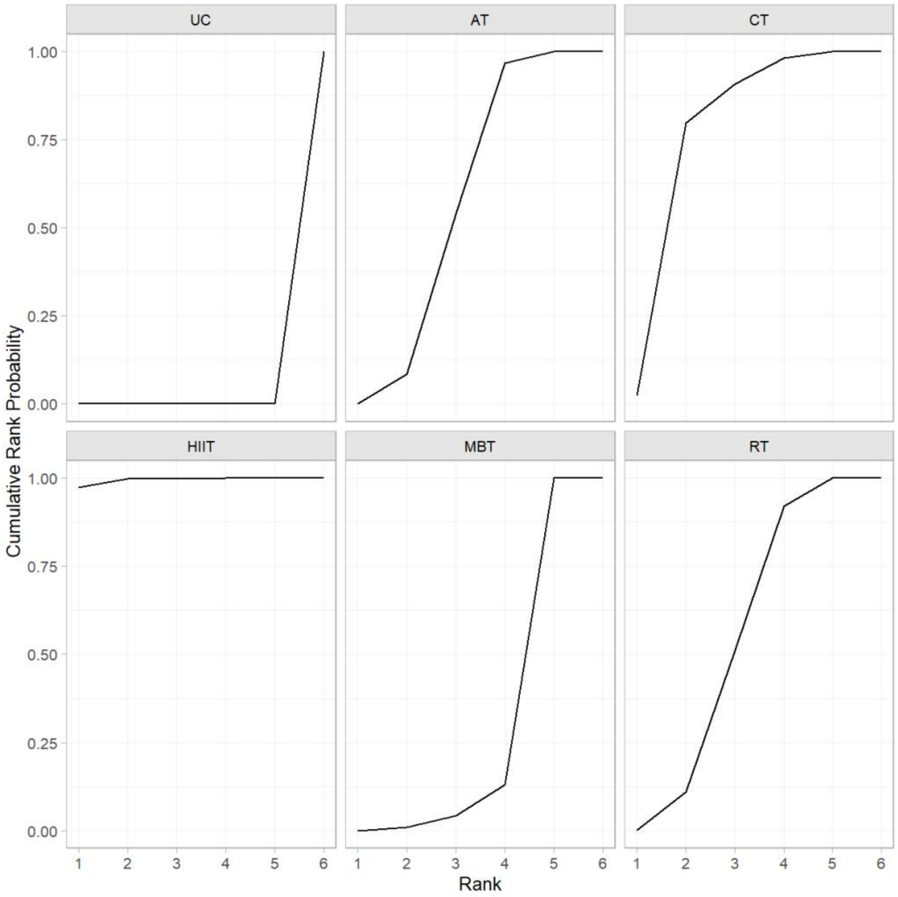

Table S5.1: SUCRA of the effects of different exercise modality on HbA1c.

	SUCRA	PrBest	MeanRank
**HIIT**	99	97	1.03
**CT**	74	3	2.29
**AT**	52	0	3.41
**RT**	51	0	3.46
**MBT**	24	0	4.82
**UC**	0	0	6

Appendix 6: Forest Plot of Pairwise Comparisons from Meta-Analysis

Figure S6.1: Forest Plot of Pairwise Comparisons (AT: CT) from Meta-Analysis

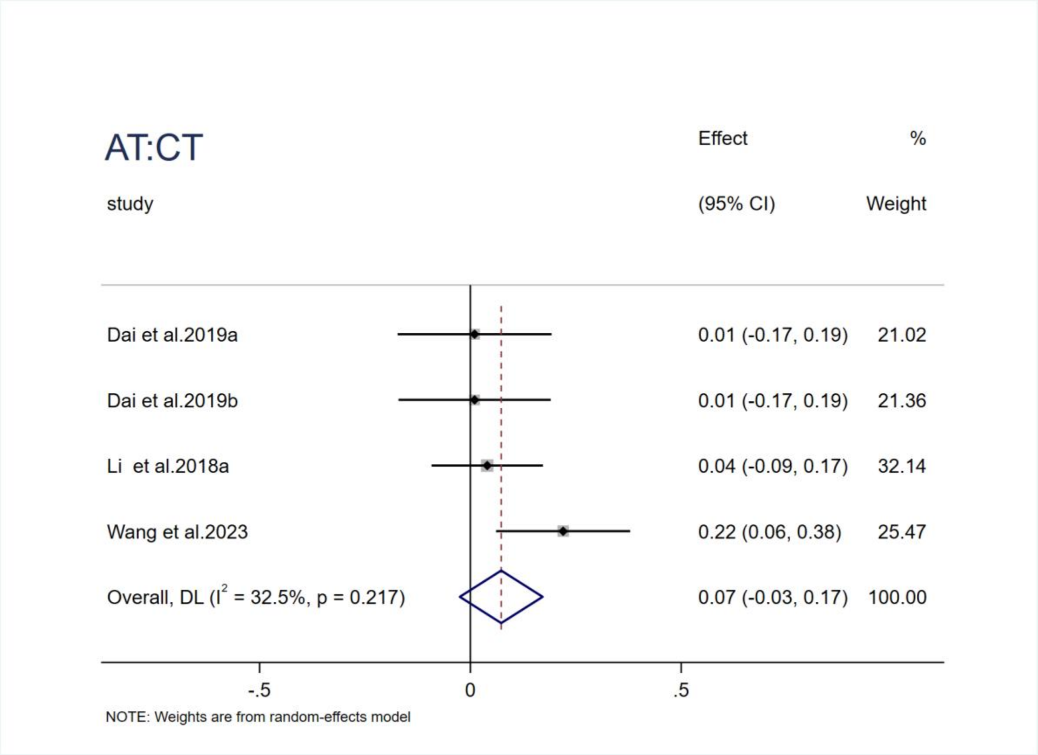

Figure S6.2: Forest Plot of Pairwise Comparisons(AT: HIIT) from Meta-Analysis

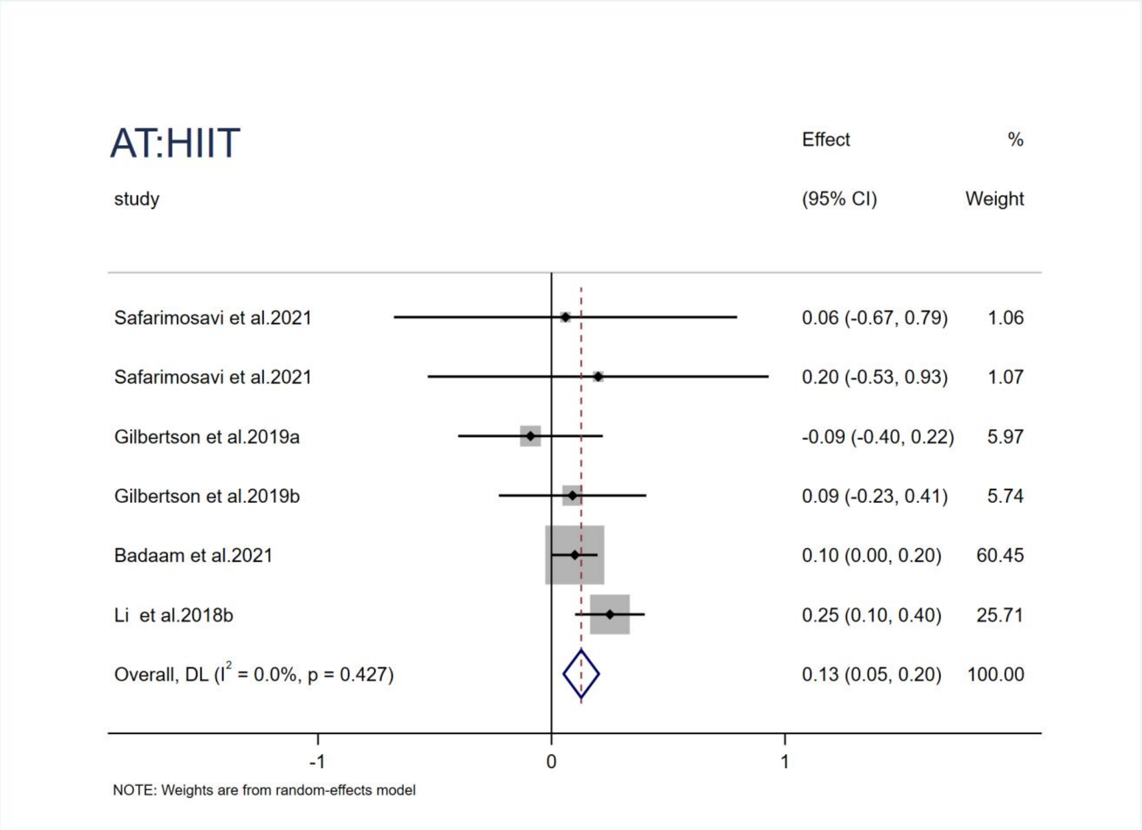

Figure S6.3: Forest Plot of Pairwise Comparisons (AT: MBT) from Meta-Analysis

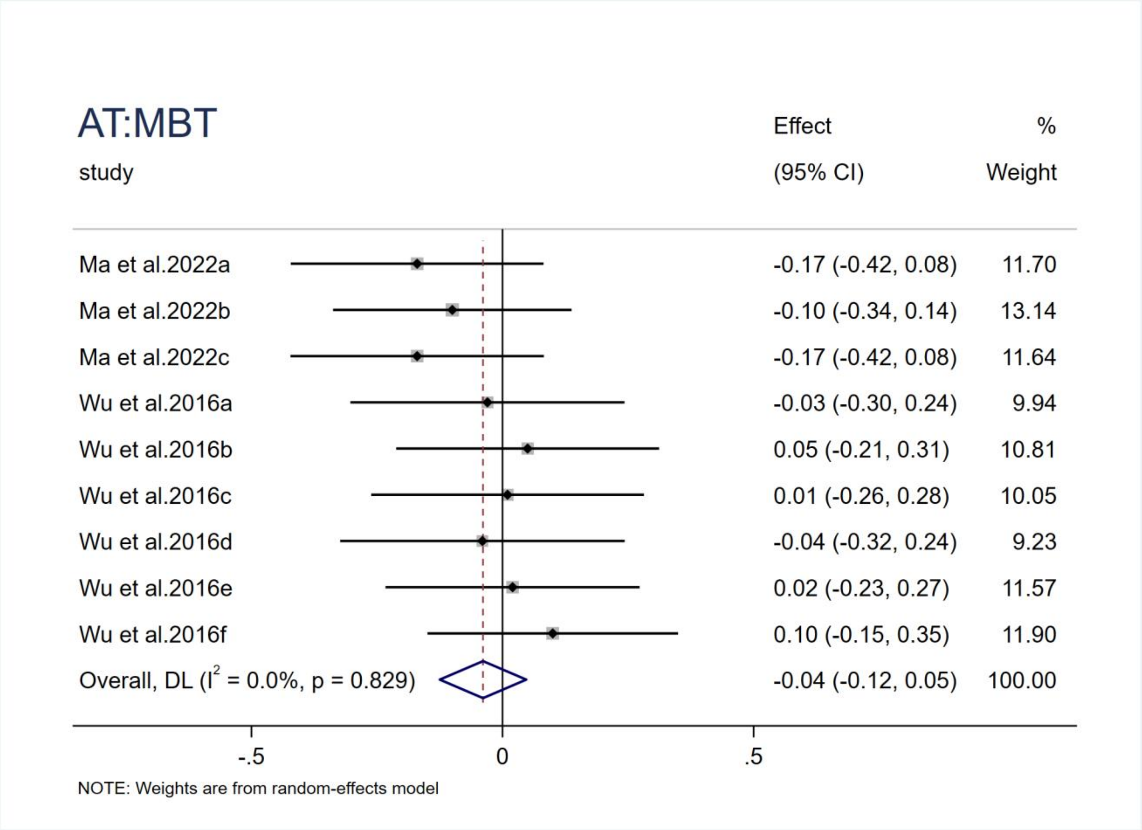

Figure S6.4: Forest Plot of Pairwise Comparisons (AT: RT) from Meta-Analysis

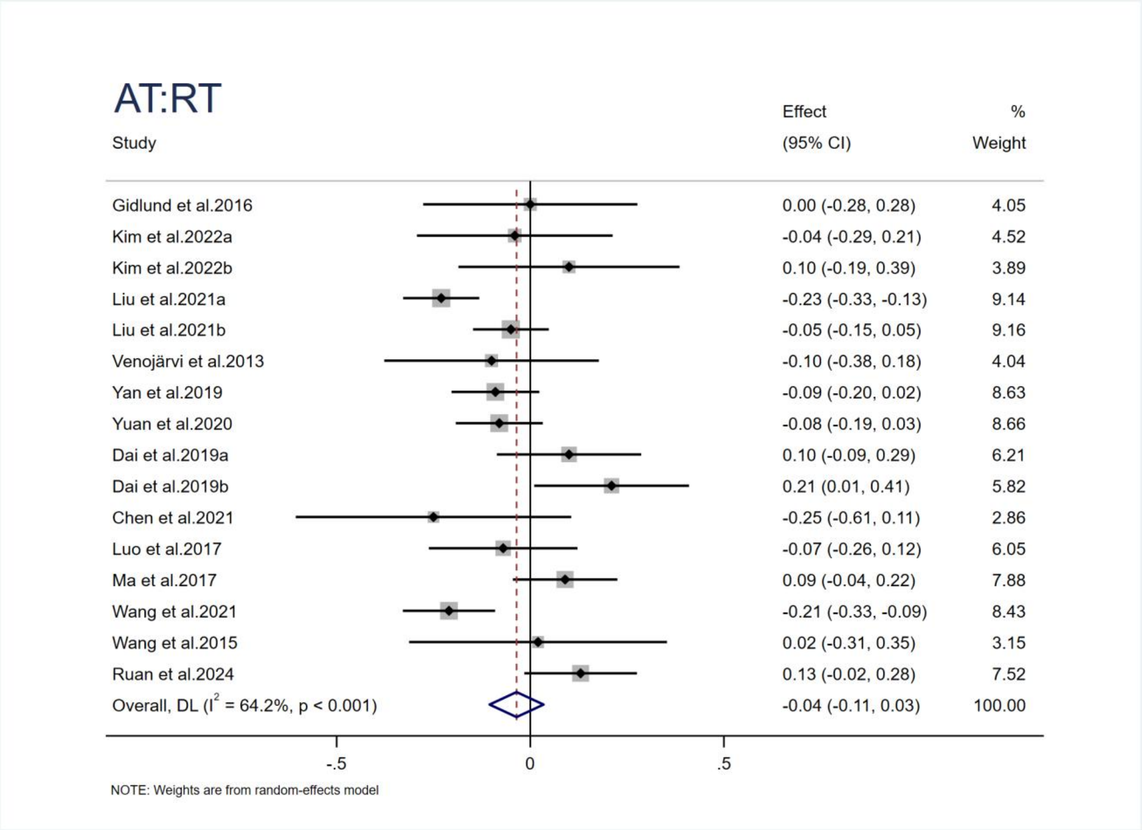

Figure S6.5: Forest Plot of Pairwise Comparisons (AT: UC) from Meta-Analysis

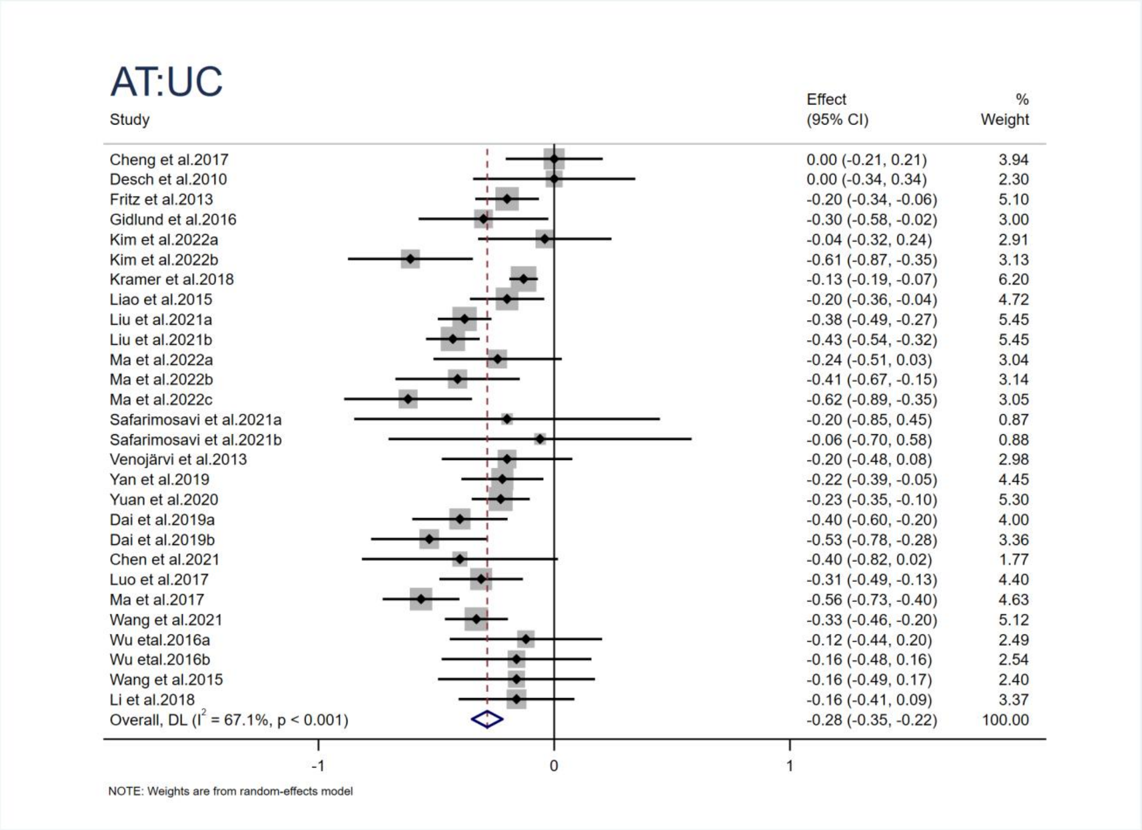

Figure S6.6: Forest Plot of Pairwise Comparisons (CT: HIIT) from Meta-Analysis

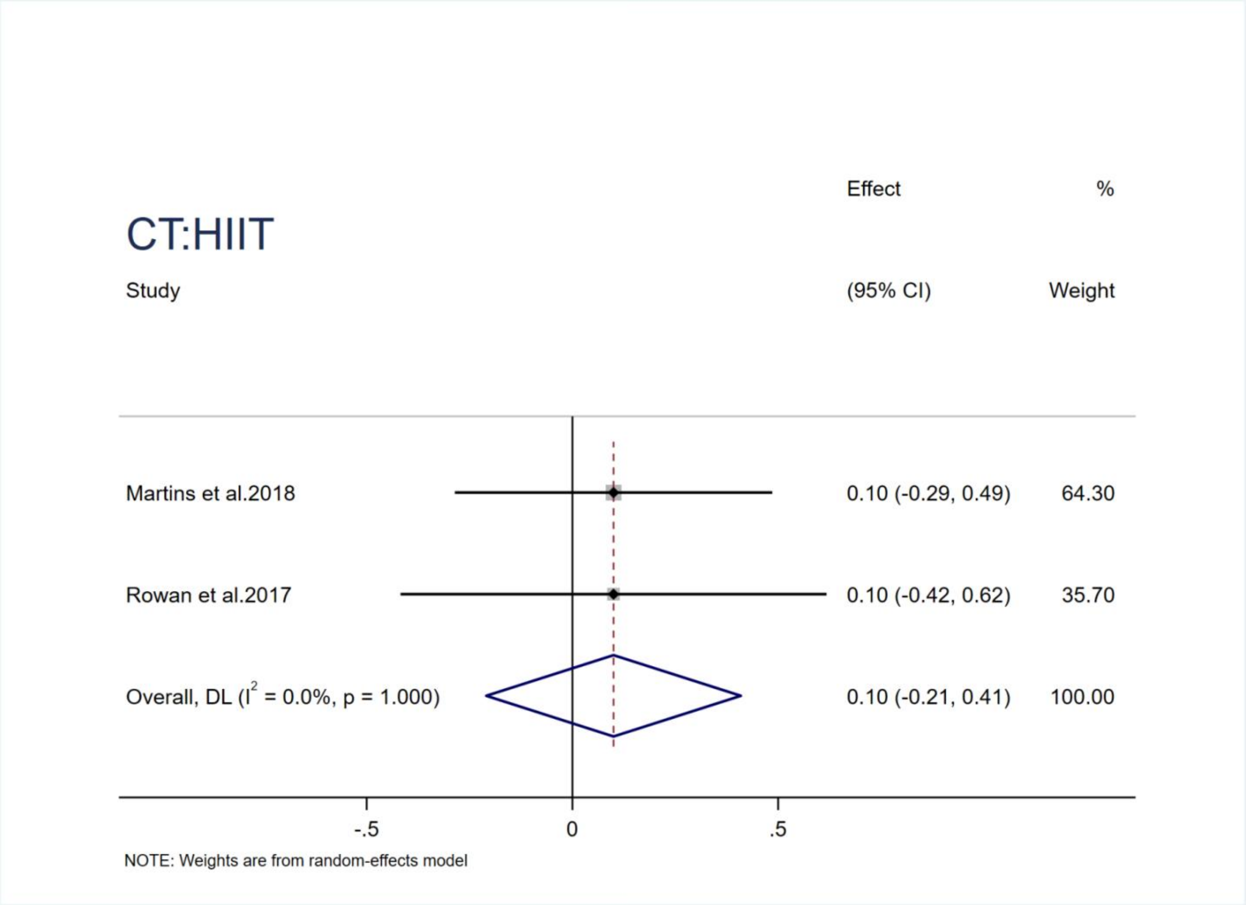

Figure S6.7: Forest Plot of Pairwise Comparisons (CT: HIIT) from Meta-Analysis

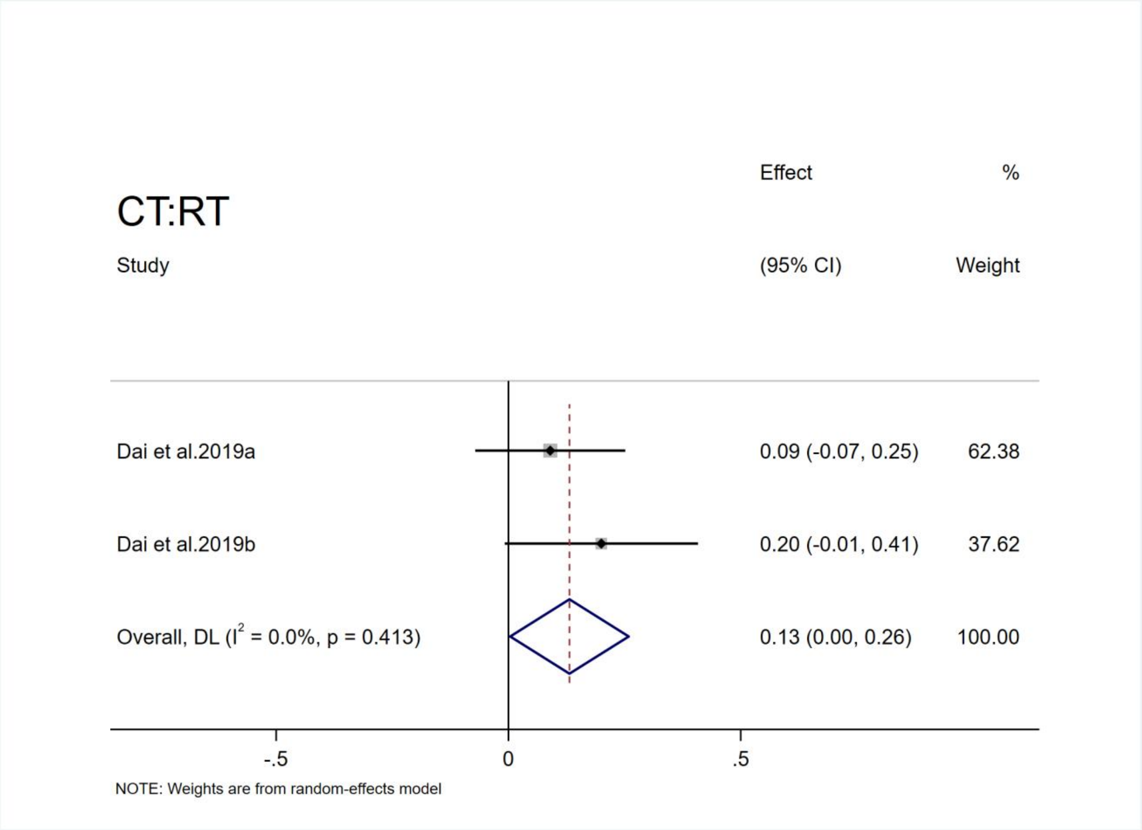

Figure S6.8: Forest Plot of Pairwise Comparisons (CT: UC) from Meta-Analysis

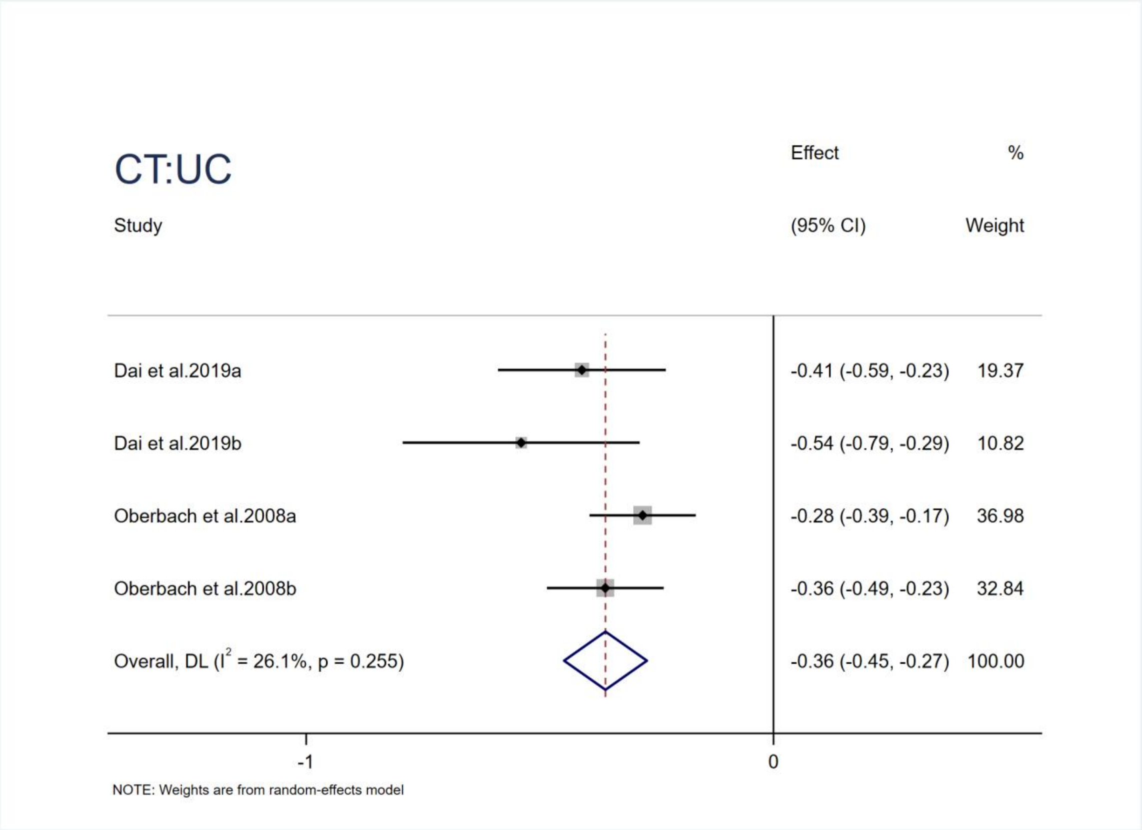

Figure S6.9: Forest Plot of Pairwise Comparisons (HIIT: UC) from Meta-Analysis

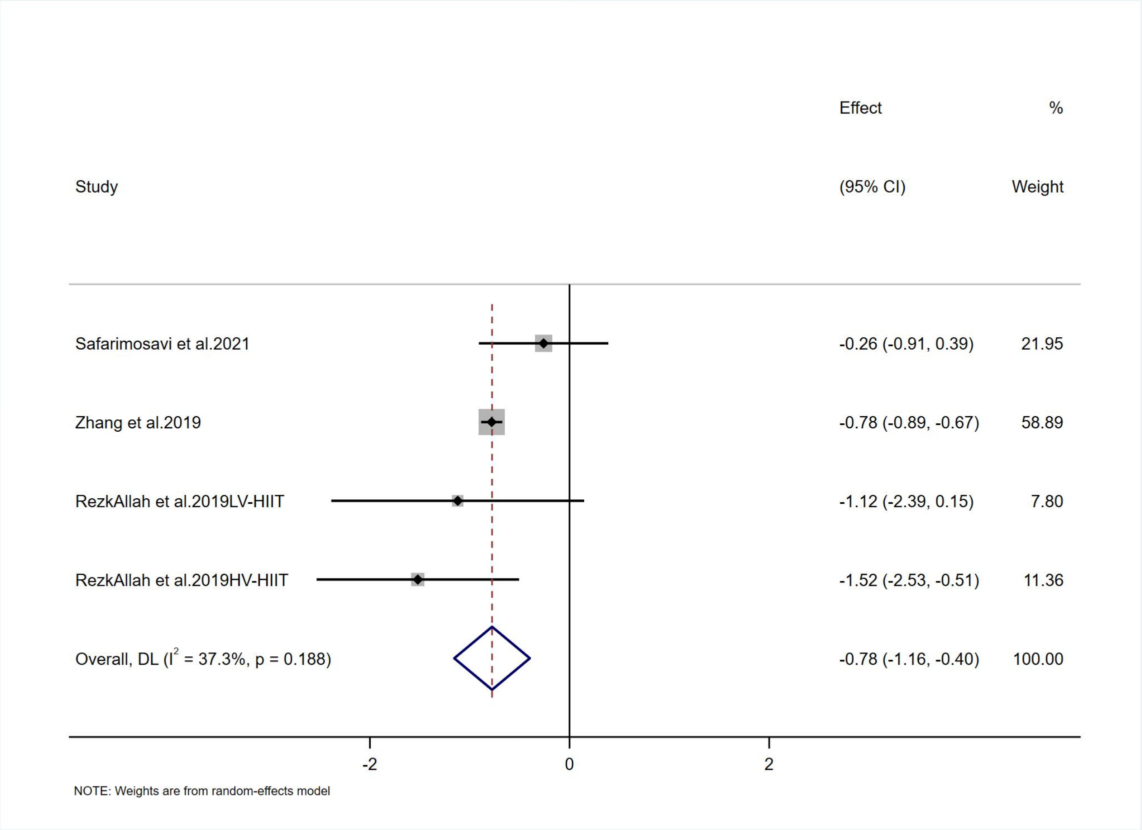

Figure S6.10: Forest Plot of Pairwise Comparisons (MBT: UC) from Meta-Analysis

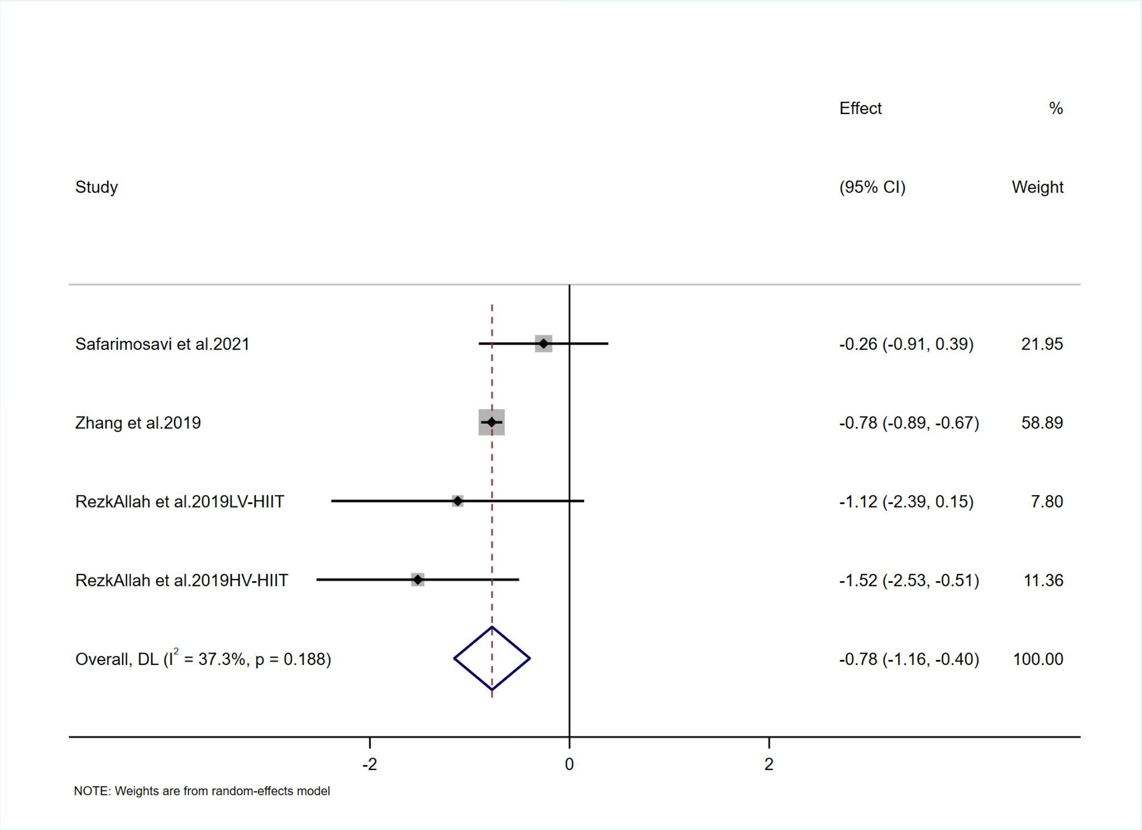

Figure S6.11: Forest Plot of Pairwise Comparisons (RT: UC) from Meta-Analysis

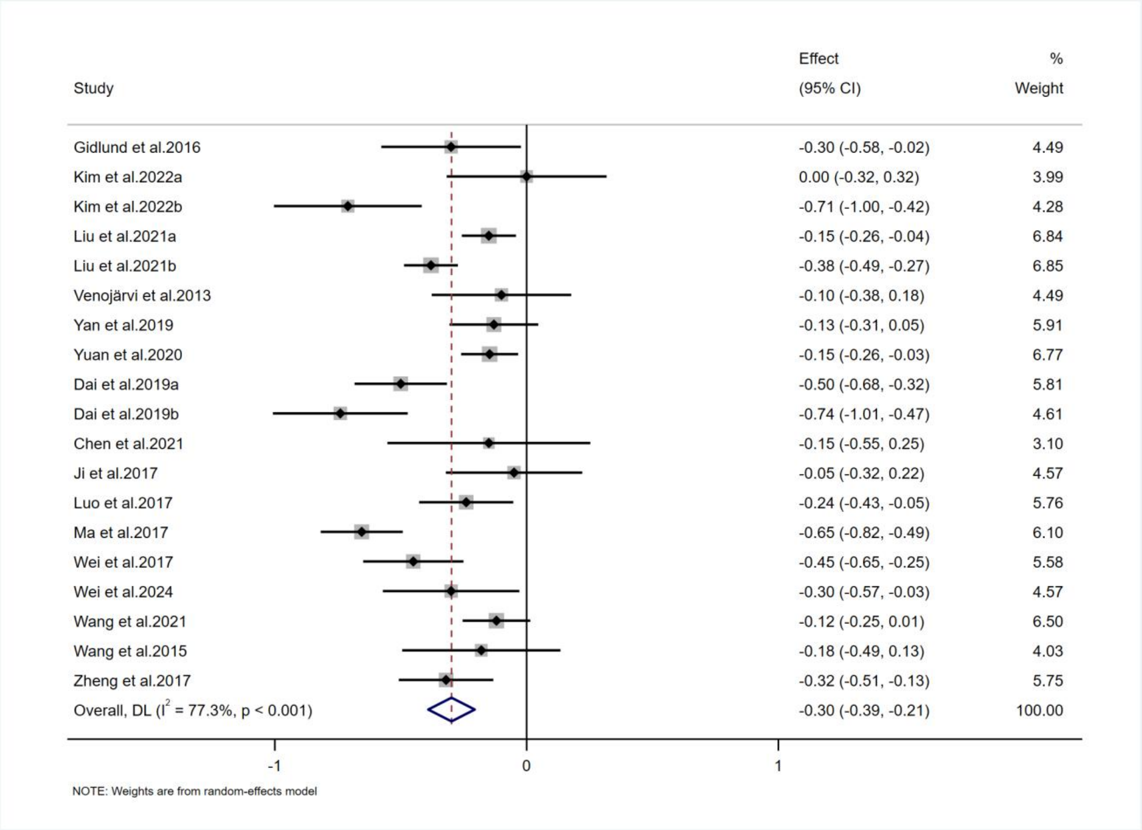

Appendix 7: Dose-response relationship between exercise dose and HbA1c reduction.

Figure S7.1: Dose-response relationship between total exercise (weekly) and HbA1c levels in prediabetes for each type of exercise.

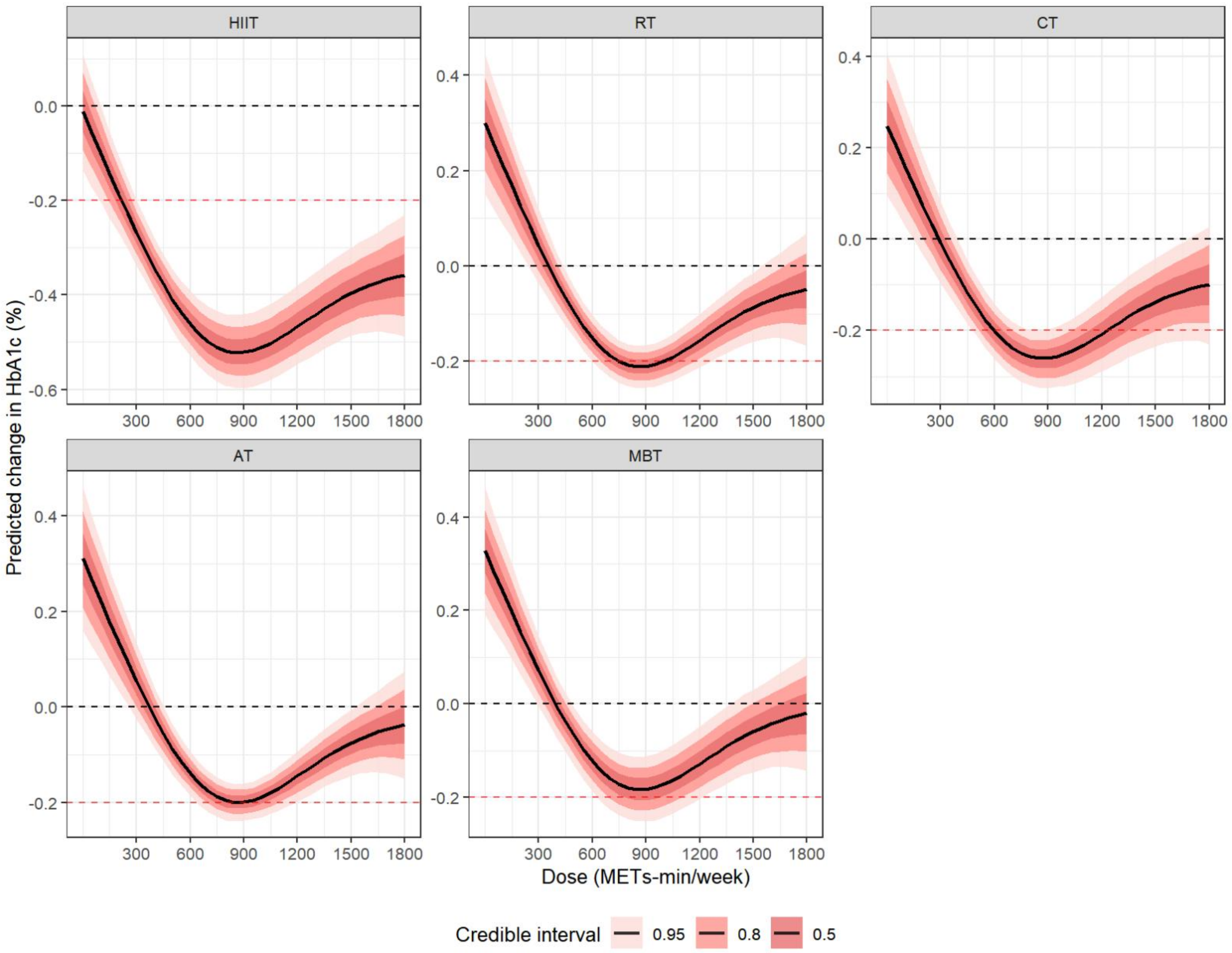

Figure S7.2: Dose-response of overall physical activity contour plot according to BMI category

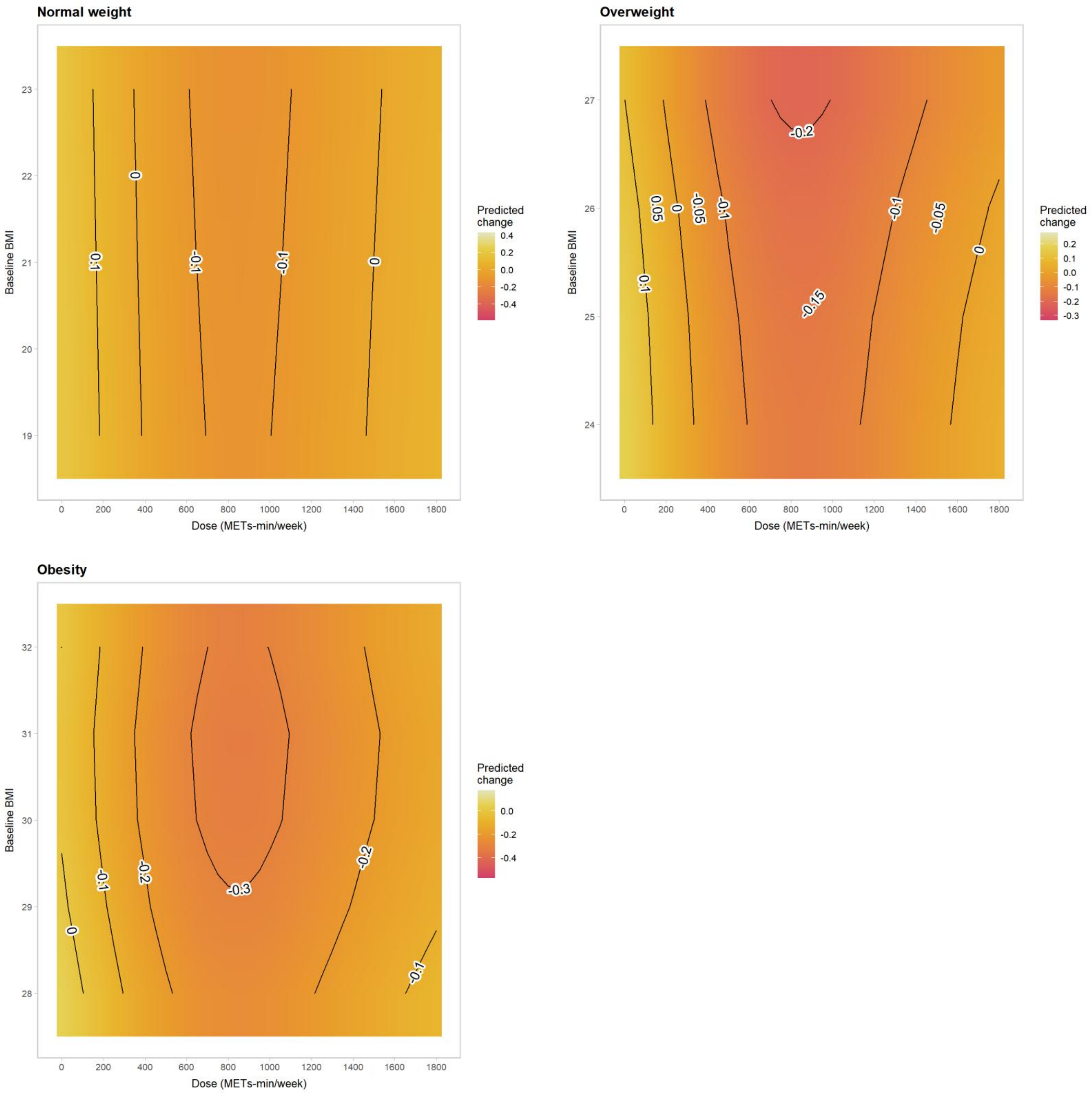

Table S7.1: Minimal effective and optimal exercise doses by single-unit BMI categories.

ADA category	Baseline BMI	Dose<br>Optimal or Minimal	MET min/week	MCFB % HbA_{1c} (95% CrI)
Normal weight	19	Optimal*	850	-0.262(-0.456, -0.0670)
		Minimal*	550	-0.213(-0.408, -0.0176)
	20	Optimal*	850	-0.247(-0.411, -0.0828)
		Minimal*	500	-0.180(-0.345, -0.0145)
	21	Optimal*	850	-0.231(-0.366, -0.097)
		Minimal*	450	-0.144(-0.280, -0.00845)
	22	Optimal*	850	-0.216(-0.322, -0.110)
		Minimal*	450	-0.129(-0.236, -0.0213)
	23	Optimal*	850	-0.201(-0281, -0.120)
		Minimal*	400	-0.0911(-0.173, -0.00950)
Overweight	24	Optimal*	850	-0.188(-0.250, -0.127)
		Minimal*	400	-0.0787(-0.141, -0.0163)
	25	Optimal*	850	-0.186(-0.238, -0.133)
		Minimal*	350	-0.0527(-0.105, -0.0000169)
	26	Optimal*	850	-0.202(-0.252,-0.152)
		Minimal*	350	-0.0690(-0.119, -0.0192)

	27	Optimal*	850	-0.239(-0.289, -0.189)
		Minimal*	250	-0.0541(-0.102, -0.00604)
Obesity	28	Optimal**	850	-0.288(-0.342, -0.234)
		Minimal**	650	-0.268(-0.326, -0.209)
	29	Optimal**	850	-0.335(-0.402, -0.269)
		Minimal**	500	-0.267(-0.322, -0.212)
	30	Optimal**	850	-0.367(-0.448, -0.285)
		Minimal**	500	-0.299(-0.382, -0.217)
	31	Optimal**	850	-0.371(-0.465, -0.278)
		Minimal**	500	-0.304(-0.399, -0.210)
	32	Optimal**	850	-0.345(-0.445,-0.245)
		Minimal**	600	-0.311(-0.412, -0.210)

MCFB % HbA1c. Mean Change from Baseline % HbA1c.
*Indicates that MCFB is statistically significant but not clinically meaningful because the 95% Cr includes values greater than –0.20%.
**Indicates that MCFB % HbA1c (95% CrI) is clinically and statistically significant.

Figure S7.3: Dose-response relationship between total exercise (weekly) and HbA1c levels in prediabetes categorized by BMI and exercise type.

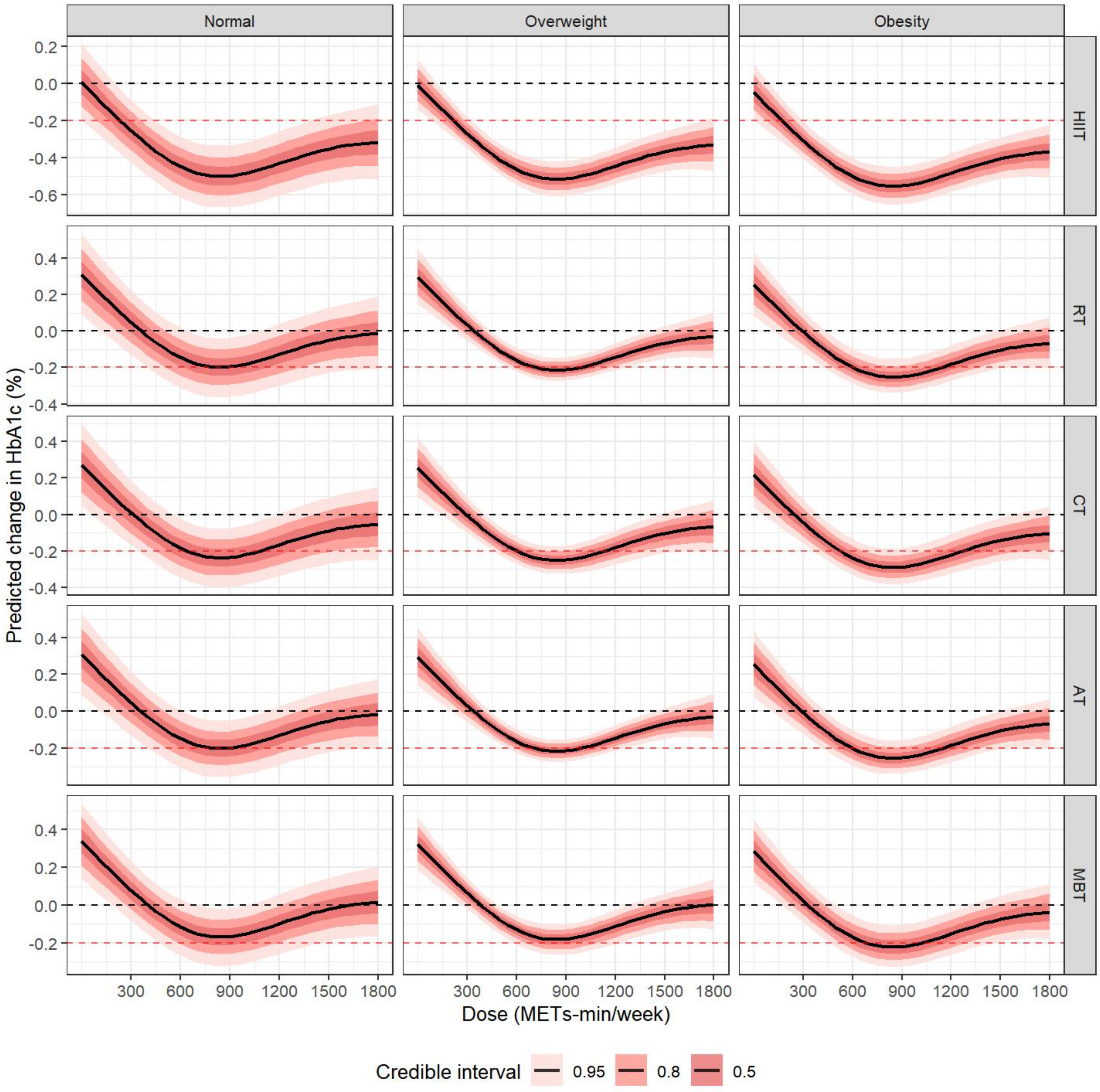

Figure S7.4: Dose-response contour plots of HbA1c levels by BMI category and exercise type.

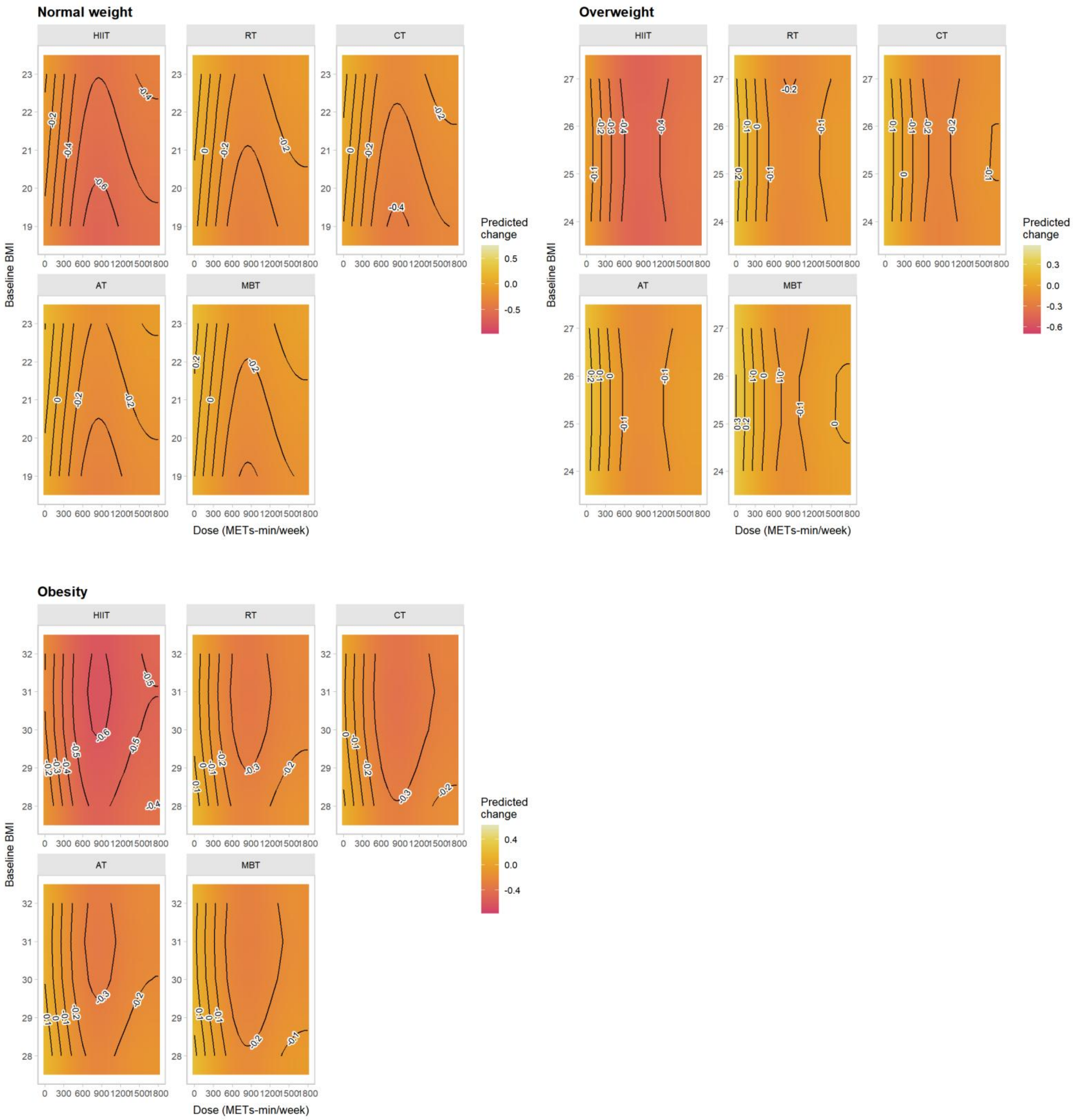

Table S7.2: Minimal effective and optimal exercise doses by BMI category and exercise type.

BMI Category	Type of exercises	Minimal Dose (METs min/week)	MCFB % HbA_{1c} (95% CrI)	Optimal Dose (METs min/week)	MCFB % HbA_{1c} (95% CrI)	Code	Mets	Recommended accumulation (min/weeks)	
								Minimum	Optimal
Normal weight	HIIT	450**	-0.364(0.524,0.205)	850**	-0.501(-0.663,-0.339)	02210	7	64	121
						02214	11	41	77
	CT	550*	-0.16(-0.317,-0.003)	850*	-0.237(-0.393,-0.081)	02054 17346	3.2	172	266
						02052 17358	4.9	112	173
						02050 17364	6.4	86	133
	RT	650*	-0.165(-0.322,-0.008)	850*	-0.198(-0.354,-0.043)	02054	3.5	186	243
						02052	5	130	170
						02050	6	108	142
	AT	650*	-0.167(-0.314,-0.019)	850*	-0.2(-0.346,-0.054)	17346	2.8	232	304
						17358	4.8	135	177
						17364	6.8	96	125
	MBT	700*	-0.15(-0.295,-0.006)	850*	-0.169(-0.314,-0.023)	15670	3.3	212	258
						02160	4	175	213

						15674	6	117	142
Overweight	HIIT	350**	-0.307(-0.389,-0.225)	850**	-0.515(-0.606,-0.42)	02210	7	50	121
						02214	11	32	77
	CT	450*	-0.114(-0.195,-0.034)	850*	-0.251(-0.327,-0.175)	02054 17346	3.2	141	266
						02052 17358	4.9	92	173
						02050 17364	6.4	70	133
	RT	450*	-0.076(-0.146,-0.005)	850*	-0.212(-0.276,-0.148)	02054	3.5	129	243
						02052	5	90	170
						02050	6	75	142
	AT	450*	-0.078(-0.148,-0.007)	850*	-0.214(-0.273,-0.155)	17346	2.8	161	304
						17358	4.8	94	177
						17364	6.8	66	125
	MBT	500*	-0.077(-0.15,-0.005)	850*	-0.182(-0.26,-0.103)	15670	3.3	152	258
						02160	4	125	213
						15674	6	83	142
Obesity	HIIT	300**	-0.306(-0.405,-0.206)	850**	-0.553(-0.651,-0.452)	02210	7	43	121
						02214	11	27	77

	CT	400*	-0.118(-0.226,-0.01)	850**	-0.289(-0.377,-0.201)	02054 17346	3.2	125	266
						02052 17358	4.9	82	173
						02050 17364	6.4	63	133
	RT	450*	-0.114(-0.212,-0.016)	850*	-0.25(-0.339,-0.161)	02054	3.5	129	243
						02052	5	90	170
						02050	6	75	142
	AT	450*	-0.116(-0.216,-0.015)	850*	-0.252(-0.341,-0.163)	17346	2.8	161	304
						17358	4.8	94	177
						17364	6.8	66	125
	MBT	500*	-0.115(-0.223,-0.008)	850*	-0.22(-0.33,-0.111)	15670	3.3	152	258
						02160	4	125	213
						15674	6	83	142

HIIT, High-Intensity Interval Training; CT; Combined Training; AT; Aerobic Training, RT, Resistance Training; MBT, Mind-Body Training.
MCFB % HbA1c. Mean Change from Baseline % HbA1c.
*Indicates that MCFB is statistically significant but not clinically meaningful because the 95% Cr includes values greater than –0.20%.
**Indicates that MCFB % HbA1c (95% CrI) is clinically and statistically significant.

Figure S7.5: Dose-response relationship between exercise intensity and HbA1c levels.

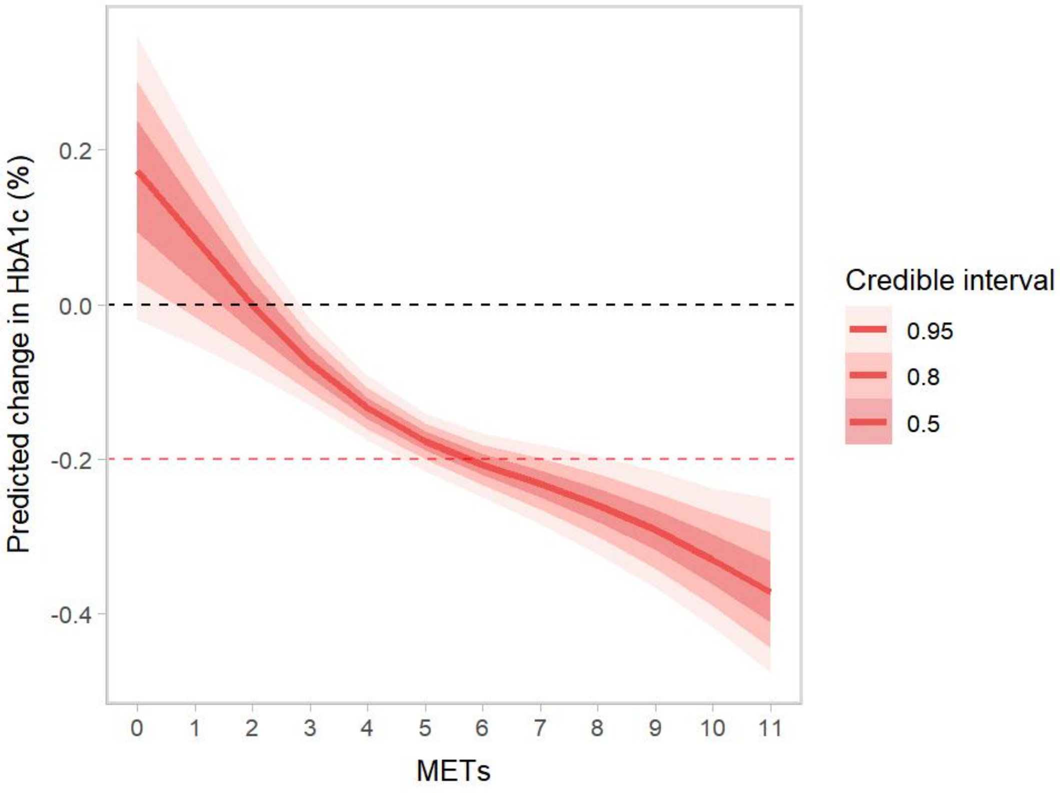

Appendix 8: CINeMA Assessment

We use the CINeMA framework to assess evidence certainty, evaluating each network estimate based on the following criteria:

- **Within study bias:** We classified the overall risk of bias for each study as low risk of bias, the risk of bias as moderate when none of the four assessed risk of bias items were rated as high risk, and the risk of bias as high when one or both items were rated as high risk. See **Appendix 4** for the bias assessment.
- **Reporting bias:** We judged it visually by a funnel plot **(Appendix 9)**.
- **Indirectness:** Transferability assumptions were assessed by reporting baseline glycated hemoglobin levels in the included study population and by comparing age and BMI at baseline concordance between groups.
- **Imprecision:** We use the CINeMA website to grade the accuracy of each comparison.
- **Heterogeneity:** We assessed the degree of worry by comparing clinical reasoning based on 95% confidence intervals (CIs) while applying the same clinical reasoning framework as for inaccuracy. In particular, we judged the consistency of our findings based on the confidence and prediction intervals associated with clinically important effect sizes. And we used the same thresholds of clinical significance as described above and followed the recommendations automatically provided by CINeMA (https://cinema.ispm.unibe.ch/).
- **Inconsistency:** For inconsistency, we looked at the results for node splitting **(Appendix 5)** and we saw major problems when $p<0.10$, but otherwise no problems.

Figure S8.1: Risk of bias contribution by intervention group in HbA1c

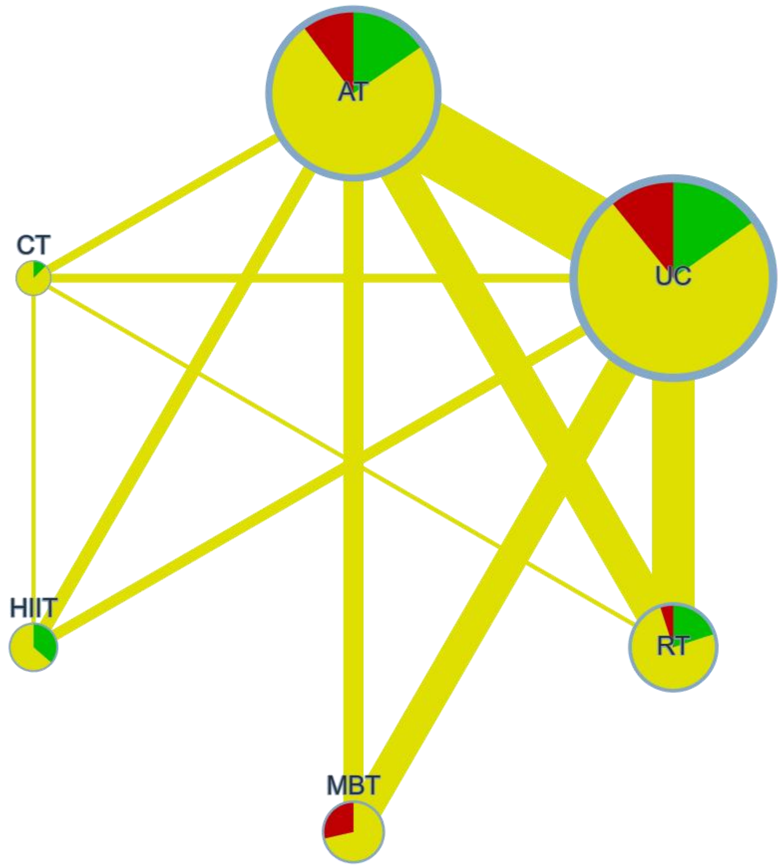

Figure S8.2: Overall risk of bias by treatment comparison in HbA1c

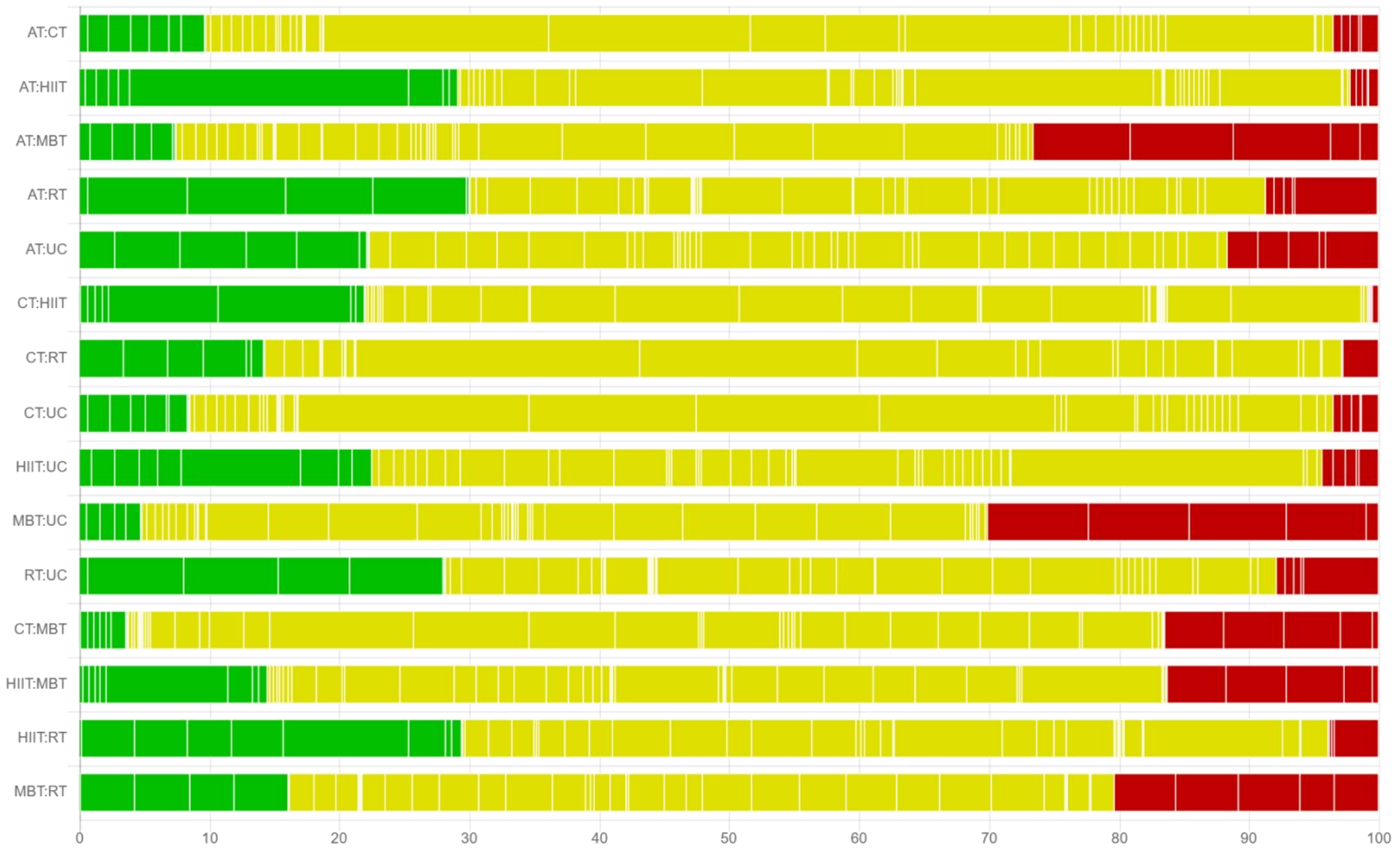

Table S8.1: Transitivity (Indirectness) Assessment

	Baseline variable (Mean ± SD)	
Intervention	Age(year)	HbA1c (%)
AT	57.17±8.22	5.91±0.21
RT	60.44±5.52	5.88±0.23
CT	57.59±4.23	5.93±0.19
HIIT	43.85±6.03	6.04±0.25
MBT	58.99±5.46	6.01±0.18
UC	56.82±5.94	5.93±0.22

Appendix 9: Funnel plots

Figure S9.1: Funnel plot of HbA1c

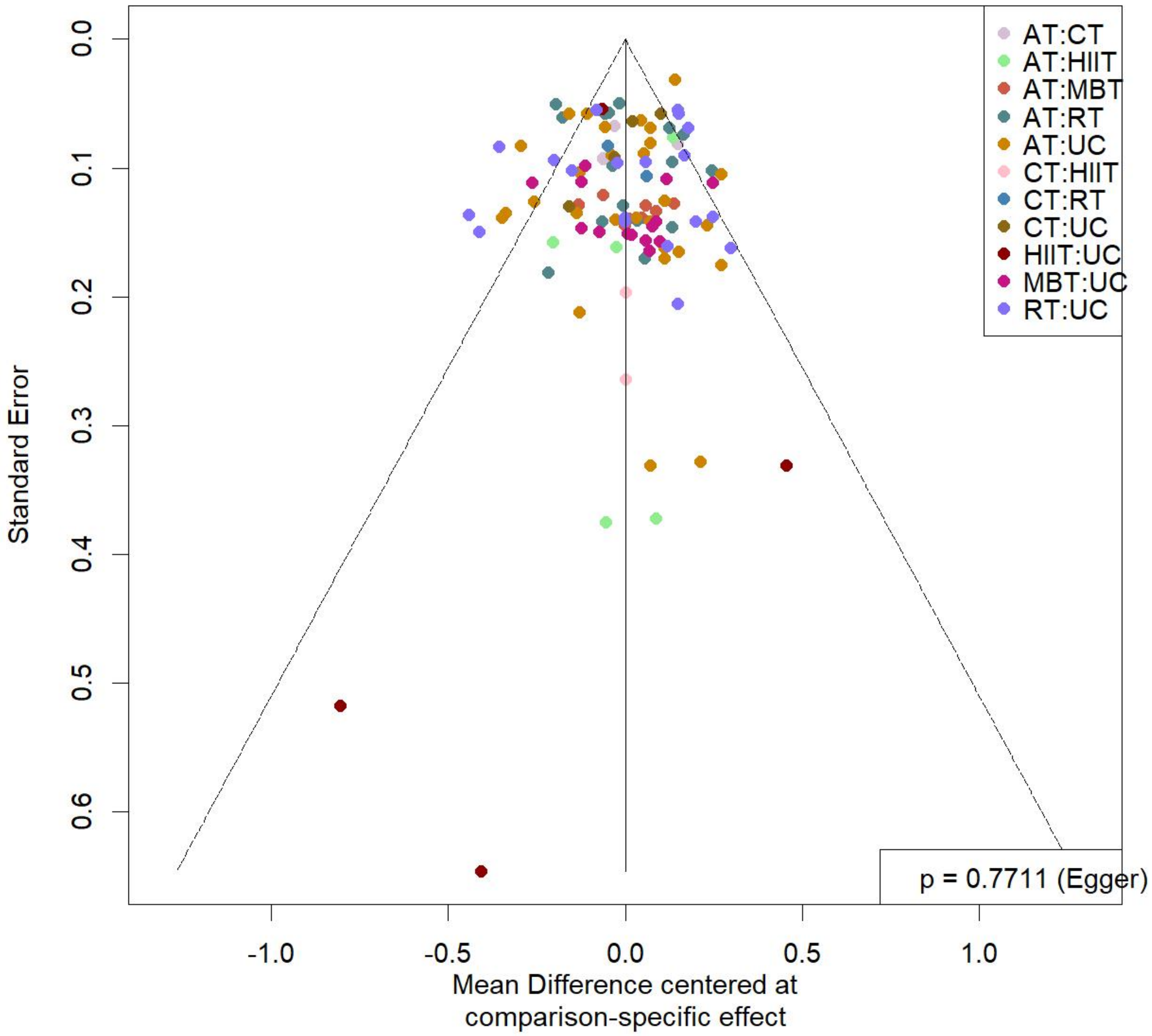

Appendix10: Network Meta-Regression

Table S10.1 Outcome of Network Meta-Regression

Covariate	HbA1c
Publish Year	-0.075714(-0.173267,0.01995)
Sample Size	-0.024499(-0.11747,0.06748)
Mean Age	-0.009527(-0.15516,0.13537)
Percentage of Male	0.053842(-0.05398,0.16156)
Baseline BMI	-0.004162(-0.11608,0.10404)
Exercise period	-0.12827(-0.21592,-0.04062)
**Exercise Frequency**	**0.103361(0.002408,0.20468)**
Time per Session	-0.009486(-0.13961,0.11782)
Baseline HbA1c	-0.050012(-0.13887,0.03953)

Crl:credible interval;*:significant influence factors, 95% Crl does not contain zero

Appendix11: Sensitivity analysis

11.1 Publication year

When the model was adjusted for centering value of publish year 2018, the hierarchy from the unadjusted model retained.

Figure S11.1 presents the impact of various exercise interventions on overall symptom changes after adjusting for the publication year 2018. It compares and ranks different exercise types against the Usual care (UC) based on the mean difference (MD). The interventions crossing the y-axis show no significant difference compared to the control group.

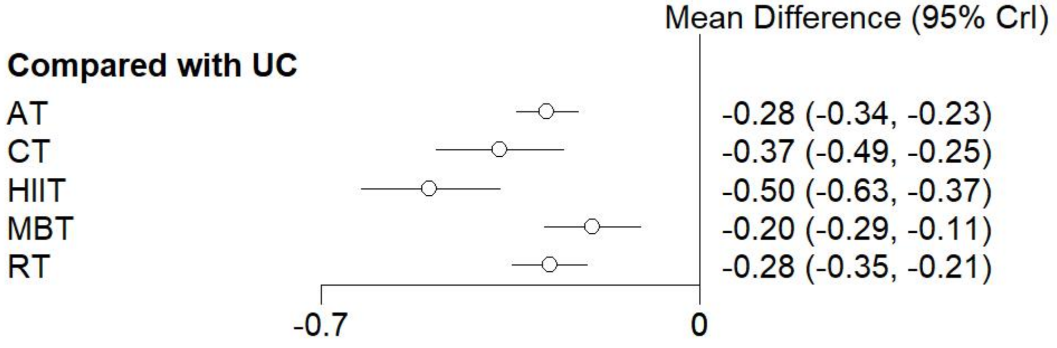

11.2 Sample size

When the model was adjusted for centering value of sample size 33, the hierarchy from the unadjusted model retained.

Figure S11.2 presents the impact of various exercise interventions on overall symptom changes after adjusting for the sample size 33. It compares and ranks different exercise types against the Usual care (UC) based on the mean difference (MD). The interventions crossing the y-axis show no significant difference compared to the control group.

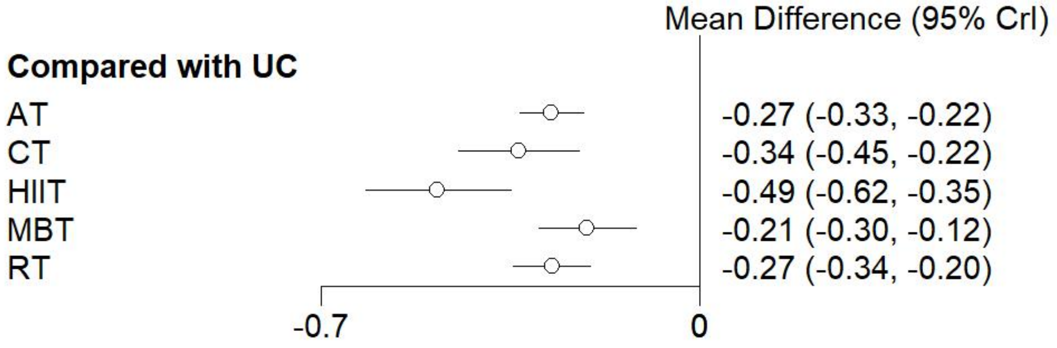

11.3 Mean age

When the model was adjusted for centering value of mean age 56, the hierarchy from the unadjusted model retained.

Figure S11.3 presents the impact of various exercise interventions on overall symptom changes after adjusting for the mean age of 56. It compares and ranks different exercise types against the Usual care (UC) based on the mean difference (MD). The interventions crossing the y-axis show no significant difference compared to the control group.

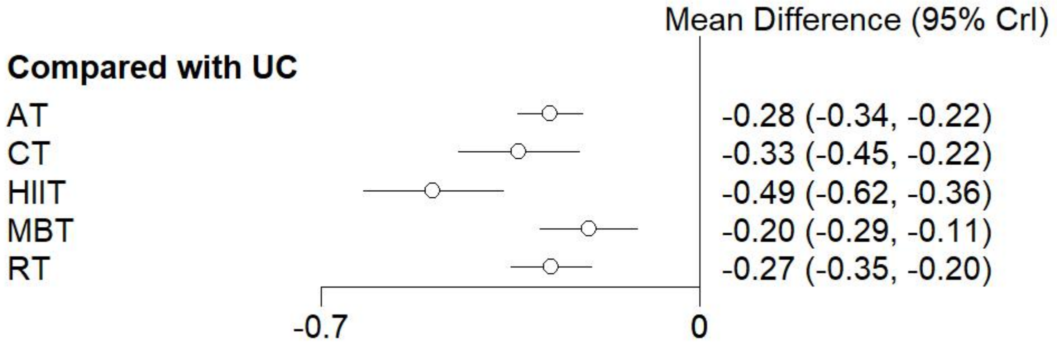

11.4 Percentage of Male

When the model was adjusted for centering value of male's percentage 44%, the hierarchy from the unadjusted model retained.

Figure S11.4 presents the impact of various exercise interventions on overall symptom changes after adjusting for the male's percentage of 44%. It compares and ranks different exercise types against the Usual care (UC) based on the mean difference (MD). The interventions crossing the y-axis show no significant difference compared to the control group.

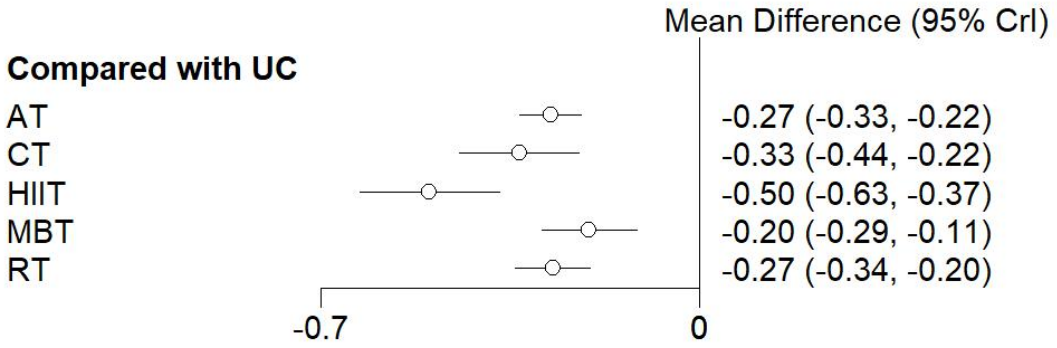

11.5 Baseline BMI

When the model was adjusted for centering value of BMI 27, the hierarchy from the unadjusted model retained.

Figure S11.5 presents the impact of various exercise interventions on overall symptom changes after adjusting for BMI 27. It compares and ranks different exercise types against the Usual care (UC) based on the mean difference (MD). The interventions crossing the y-axis show no significant difference compared to the control group.

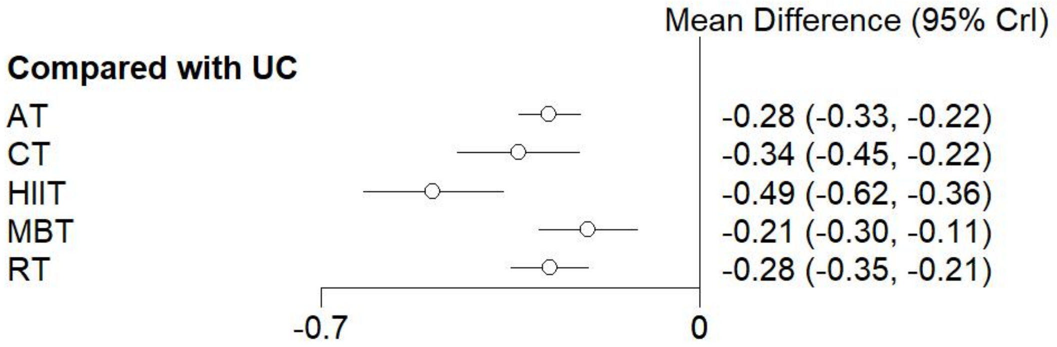

11.6 Exercise period

When the model was adjusted for centering value of exercise period 24 weeks, the hierarchy from the unadjusted model retained.

Figure S11.6 presents the impact of various exercise interventions on overall symptom changes after adjusting for the exercise period of 24 weeks. It compares and ranks different exercise types against the Usual care (UC) based on the mean difference (MD). The interventions crossing the y-axis show no significant difference compared to the control group.

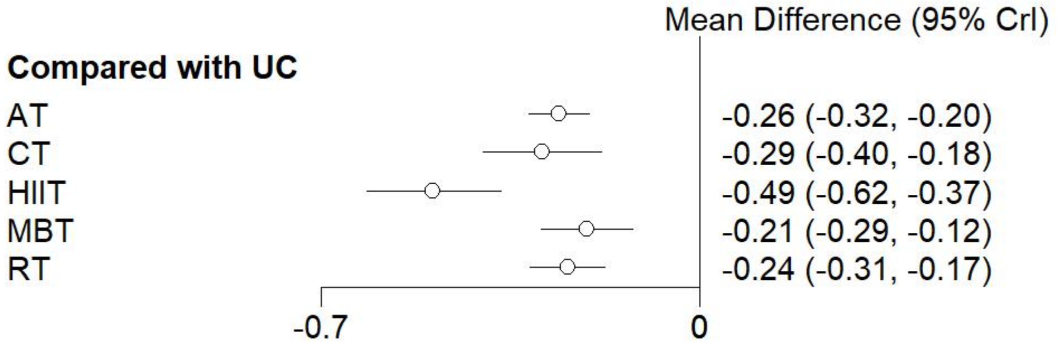

11.7 Exercise frequency

When the model was adjusted for centering value of exercise frequency (4 times per week), the hierarchy from the unadjusted model retained.

Figure S11.7 presents the impact of various exercise interventions on overall symptom changes after adjusting for the exercise frequency of 4 times per week. It compares and ranks different exercise types against the Usual care (UC)based on the mean difference (MD). The interventions crossing the y-axis show no significant difference compared to the control group.

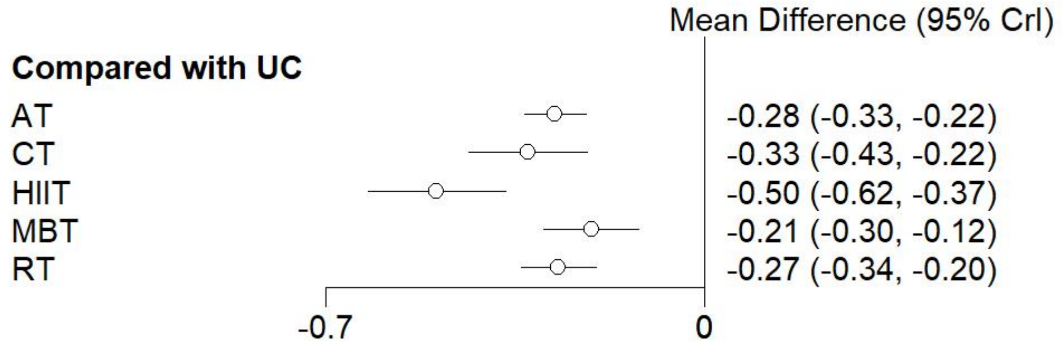

11.8 Time per session

When the model was adjusted for centering value of time per session (38 minutes), the hierarchy from the unadjusted model retained.

Figure S11.8 presents the impact of various exercise interventions on overall symptom changes after adjusting for the time per session of 38 minutes. It compares and ranks different exercise types against the Usual care (UC) based on the mean difference (MD). The interventions crossing the y-axis show no significant difference compared to the control group.

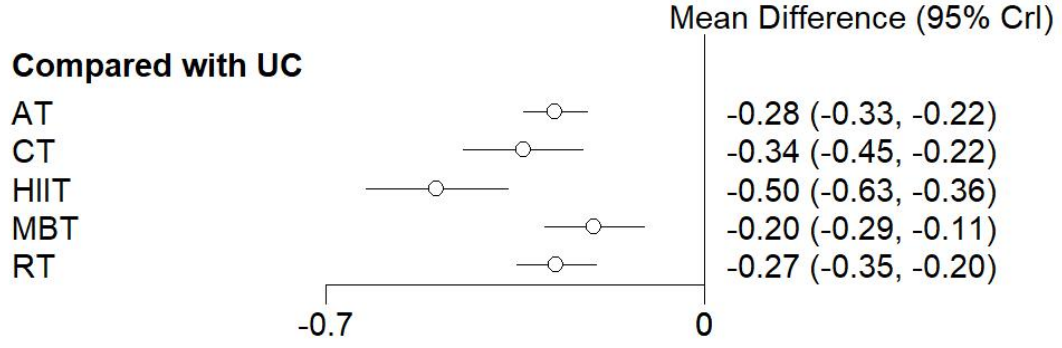

11.9 Baseline HbA1c

When the model was adjusted for centering value of Baseline HbA1c (5.93%), the hierarchy from the unadjusted model retained.

Figure S11.9 presents the impact of various exercise interventions on overall symptom changes after adjusting for the time per week of 5.93%. It compares and ranks different exercise types against the Usual care (UC) based on the mean difference (MD). The interventions crossing the y-axis show no significant difference compared to the control group.

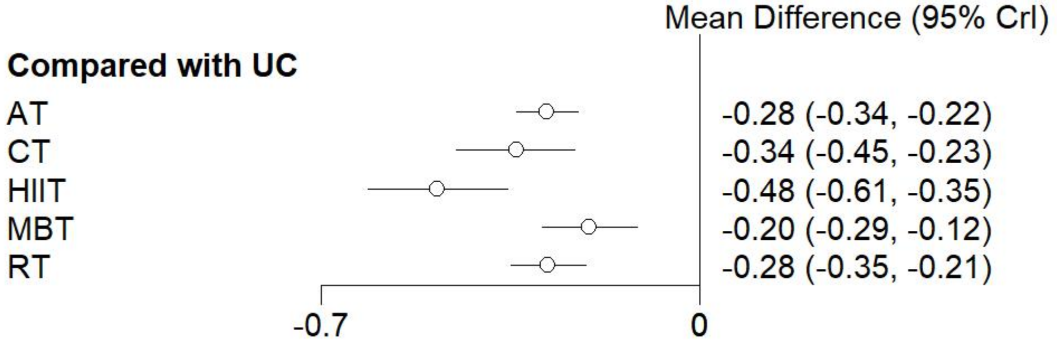

Table S11.1 Sensitivity analyses of HbA1c effect-size estimates under different assumed pre–post correlations (r = 0.3, 0.5, and 0.7)

Intervention	r = 0.3 (MD, 95% CI)	r = 0.5 (MD, 95% CI)†	r = 0.7 (MD, 95% CI)
HIIT	-0.5(-0.64,-0.36)	-0.5(-0.62,-0.36)	-0.48(-0.62,-0.34)
CT	-0.32(-0.44,-0.20)	-0.34(-0.45,-0.23)	-0.31(-0.45,-0.19)
RT	-0.28(-0.36,-0.20)	-0.28(-0.36,-0.21)	-0.28(-0.36,-0.21)
AT	-0.29(-0.35,-0.22)	-0.28(-0.33,-0.22)	-0.28(-0.34,-0.22)
MBT	-0.21(-0.31,-0.11)	-0.21(-0.29,-0.12)	-0.20(-0.30,-0.10)

† Primary analysis reported in the main text.

Table S11.2 Sensitivity analysis of HbA1c effect-size estimates after excluding high-risk-of-bias studies

Intervention	MD, 95% CI
HIIT	-0.51(-0.65,-0.37)
CT	-0.29(-0.39,-0.17)
RT	-0.24(-0.32,-0.16)
AT	-0.24(-0.31,-0.17)
MBT	-0.15(-0.27,-0.04)
