# Supplementary material for: Optimal exercise dose for glycemic control in prediabetes across different exercise types
Source: iScience. 2025 Nov 10;28(12):113980. doi: 10.1016/j.isci.2025.113980 (PMC12689185; doi:10.1016/j.isci.2025.113980)
Supplement: Methods S1. Definitions of Exercise Interventions [file mmc2.pdf]

Aerobic Training (AT) [1]: Characterized by prolonged activities that increase heart rate and respiration, primarily targeting cardiovascular endurance. Common forms include walking, running, cycling, and swimming.

Resistance Training (RT) [1]: Activities designed to enhance muscular strength, endurance, and hypertrophy by applying resistance. Typical examples include weightlifting, bodyweight exercises, and resistance band workouts.

Combined Training (CT) [1]: Refers to combining aerobic and resistance exercises within a single session or training program. Its purpose is to provide the benefits of both modalities, such as improved cardiovascular health and muscle strength.

Mind-body Training (MBT)[2]: refers to movement-based exercise modalities that integrate structured physical postures or sequences with breath regulation and mindful attentional focus to elicit psychophysiological responses. MBT is not culture-specific and includes, for example, yoga (e.g., Hatha, Iyengar), tai chi, qigong/Baduanjin, and selected Pilates-based programmes. For this review, MBT excludes non-movement mind–body interventions such as seated meditation, breathing-only practices, progressive muscle relaxation, or education-only sessions. MBT sessions are typically of low-to-moderate intensity.

High-Intensity Interval Training (HIIT) [3,4]: Defined as exercising at "near maximal" intensity, characterized by alternating between high-intensity exercise and low-intensity recovery or rest phases. The intensity is ≥75% of VO2max, VO2peak, peak power output (PPO), HRmax, HRpeak, or HRR.

References

1. Pescatello, L. S. *ACSM's Guidelines for Exercise Testing and Prescription*. (Lippincott Williams & Wilkins, 2014).

2. Blomstrand, P., Tesan, D., Nylander, E. M. & Ramstrand, N. Mind body exercise improves cognitive function more than aerobic- and resistance exercise in healthy adults aged 55 years and older – an umbrella review. *Eur Rev Aging Phys Act* **20**, 15 (2023).

3. MacInnis, M. J. & Gibala, M. J. Physiological adaptations to interval training and the role of exercise intensity. *J Physiol* **595**, 2915–2930 (2017).

4. Atakan, M. M. *et al.* Effects of high-intensity interval training (HIIT) and sprint interval training (SIT) on fat oxidation during exercise: a systematic review and meta-analysis. *Br J Sports Med* **56**, 988–996 (2022).
